# Supplementary material for: DIALOR (DIgitAL cOaching for fRailty): protocol for a single-arm mixed-methods feasibility study of a digital health coaching intervention for older people with frailty in primary care
Source: BMJ Open. 2024 May 16;14(5):e080480. doi: 10.1136/bmjopen-2023-080480 (PMC11103224; doi:10.1136/bmjopen-2023-080480)
Supplement: Author's manuscript [file bmjopen-2023-080480.draft_revisions.pdf]

BMJ Open is committed to open peer review. As part of this commitment we make the peer review history of every article we publish publicly available.

When an article is published we post the peer reviewers' comments and the authors' responses online. We also post the versions of the paper that were used during peer review. These are the versions that the peer review comments apply to.

The versions of the paper that follow are the versions that were submitted during the peer review process. They are not the versions of record or the final published versions. They should not be cited or distributed as the published version of this manuscript.

BMJ Open is an open access journal and the full, final, typeset and author-corrected version of record of the manuscript is available on our site with no access controls, subscription charges or pay-per-view fees (http://bmjopen.bmj.com).

If you have any questions on BMJ Open's open peer review process please email info.bmjopen@bmj.com

BMJ Open

DIALOR (DIgitAL cOaching for fRailty): Protocol for a single-arm mixed methods feasibility study of a digital health coaching intervention for older people with frailty in primary care.

Journal:	*BMJ Open*
Manuscript ID	bmjopen-2023-080480
Article Type:	Protocol
Date Submitted by the Author:	02-Oct-2023

Complete List of Authors:	Sait, Matthew; Bournemouth University Faculty of Health and Social Sciences, Rehabilitation and Sport Sciences<br>Christie, Rachel; Bournemouth University, Rehabilitation and Sports Sciences<br>Cox, Chantel; Bournemouth University, Nursing Science<br>Board, Michele; Bournemouth University, Nursing Science<br>Davies, Cheryl; Wessex Academic Health Science Network<br>Walker, Dawn-Marie; University of Southampton, Faculty of Health Sciences<br>Vassallo, Michael; University Hospitals Dorset NHS Foundation Trust, Older Person Medicine<br>Sadler, EA; University of Southampton, Faculty of Environmental and Life Sciences, School of Health Sciences<br>O'Sullivan, Cheryl; NHS Dorset<br>Allen-Pick, Mark; Bournemouth University, Rehabilitation and Sports Sciences<br>Moore, Patrick; The Adam Practice<br>Bradbury, Katherine; University of Southampton, Psychology<br>Murphy, Jane; Bournemouth University, Humans Sciences & Public Health

Keywords:	Aging, Health informatics < BIOTECHNOLOGY & BIOINFORMATICS, Chronic Disease, Primary Health Care

SCHOLARONE™
Manuscripts

Title	DIALOR (DIgitAL cOaching for fRailty): Protocol for a single-arm mixed methods feasibility study of a digital health coaching intervention for older people with frailty in primary care.
**Word count** (Excluding title page, abstract, references, figures, tables, or acknowledgements)	3805
**Version**	1
**Key Words**	Frailty, chronic conditions, long-term conditions, digital health interventions, health coaching
**Authors' names**	Matthew Sait (0000-0003-0226-0351), Rachel Christie, Chantel Cox (0009-0000-7241-3179), Michele Board (0000-0002-6732-0151), Cheryl Davies, Dawn-Marie Walker (0000-0003-2135-1363), Michael Vassallo, Euan Sadler (0000-0003-3827-224X), Cheryl O'Sullivan, Mark-Allen Pick, Patrick Moore (0000-0001-8279-6724), Katherine Bradbury (0000-0001-5513-7571), Jane Murphy (0000-0003-3531-5566)
**Author addresses, names, and positions**	Bournemouth University, 12 St Paul's Ln, BH8 8GP, Bournemouth, England department of rehabilitation and sports science Matthew Sait Post-Graduate Researcher and Research Project Officer, Bournemouth University Rachel Christie research assistant, department of rehabilitation and sports science, Bournemouth University Chantel Cox senior lecturer in adult nursing department of nursing science, Bournemouth University Michele Board associate professor, Wessex AHSN Cheryl Davies senior programme manager innovation adoption, University of Southampton Dawn-Marie Walker associate professor, University Hospitals Dorset Michael Vassallo consultant geriatrician, University of Southampton Euan Sadler associate professor, National Health Service (NHS) Dorset Cheryl O'sullivan advanced nurse practitioner, University Hospitals Dorset Mark-Allen Pick occupational therapist, The Adam Practice Patrick Moore GP Partner, University of Southampton Katherine Bradbury principal research fellow
**Corresponding author**	Correspondence to: J Murphy on jmurphy@bournemouth.ac.uk

For peer review only

For peer review only - http://bmjopen.bmj.com/site/about/guidelines.xhtml

Abstract (300 words)

Background

Multi-domain interventions in older adults offer the best opportunity to prevent, delay, or reverse existing symptoms in the earlier stages of frailty and improve independence but can be costly, and difficult to deliver at scale. However, digital health interventions enable personalised care and empowerment through self-management of long-term conditions, used at any time and when combined with health coaching offer potential to enhance wellbeing and facilitate achievement of health-related goals. We aim to evaluate the feasibility and acceptability of a digital health platform for long-term disease management combined with health coaching for people living with mild-moderate frailty, targeting self-identified goals – activity, nutrition, mood, enhancing social engagement and well-being.

Methods and analysis

This is a non-randomised feasibility, single group, pretest/post-test study, using qualitative and quantitative methods. The digital health coaching intervention (DIALOR - DIgitAL cOaching for fRailty) has been developed for implementation to older adults, aged 65 years or older with mild to moderate frailty and diagnosis of one or more long-term health conditions in the community. Participants will receive 12 weeks of health coaching and have access to a mobile health platform for 6 months. The primary outcome measure is the acceptability and feasibility of DIALOR along with a range of secondary outcome measures (including frailty, functioning measures, quality of life, social engagement, diet quality and self-reported indicators) collected at baseline and 6 months. The findings will inform whether a wider effectiveness trial is feasible and if so, how it should be designed.

Ethics and Dissemination

Ethical approval has been granted by the Southeast Scotland Research Ethics Committee 02 (reference: 22/SS/0064). Research findings will be disseminated in a range of different ways to engage different audiences, including publishing in open-access peer-reviewed journals, conference presentations, social media, dissemination workshop with patients, carers, and healthcare professionals and on institution websites.

Article Summary

Strengths and limitations of this study

- The study will provide new knowledge using a new digital health coaching intervention in primary care for older people with early to moderate frailty and another long-term condition with potential to inform future strategies to improve management.
- Strong involvement of stakeholders and people with the lived experience to develop and guide the study.
- Quantitative and qualitative approaches to assess study outcomes will be used to inform the design of a future larger study and whether it is feasible.

For peer review only - http://bmjopen.bmj.com/site/about/guidelines.xhtml

- Potential limitations are as a feasibility study, it will assess a pre and post-test intervention with non-randomisation of sample and to sufficiently engage hard-to reach target groups including those with poor digital literacy and low socio-economic status.

Introduction

Frailty is a multidimensional syndrome characterised by cumulative loss of reserves and accumulation of deficits (e.g., energy, physical ability, cognition, social and psychological aspects) that give rise to vulnerability and risk of adverse outcomes with advancing age (1). It is not an inevitable consequence of ageing but older adults with frailty have increased risk of poor health, including falls, fractures and functional impairment, hospitalisation, need for nursing home care, and increased mortality (2). Older people with frailty experience impairment in their activities of daily living and often report significant reduction in their quality of life (3). Furthermore, studies have shown that frailty may be related to decreased mood (4) and may be shaped by social factors such as different levels of social support or living alone (5). Frailty affects about 12% of people aged ≥ 65 years worldwide (6) and 14% of older people in the United Kingdom (UK) (7), and even higher numbers of individuals in the early stages of frailty, ranging from 19-53% (8). Frailty is an ever-present burden amongst international populations, which has been exacerbated by the impact of the Covid-19 pandemic (9) and the resultant deconditioning (10).

Whilst moderate to severe frailty has a higher risk of physical health declines, mild-moderate frailty with some loss of physiological reserve is considered to be potentially reversible (11) to a robust or stable state (12). Mild frailty is conceptualised as an intermediate stage on the Rockwood Clinical Frailty Scale (13), whereby older people experience some loss of physiological reserves but can usually recover after a negative stressor event, such as an infection or fall. This would indicate that interventions targeting earlier stages of frailty are more likely to reverse frailty decline. Moreover, there is evidence to suggest that interventions, particularly multi-domain interventions in older adults including mobility, strength, balance, nutrition, physical activity, and social support offer the best opportunity to prevent, delay, or reverse existing symptoms of frailty and improve independence (14, 15, 16, 17). Most multi-component interventions incorporate involvement from a multidisciplinary team of health and social care professionals meaning they can be costly, and difficult to deliver at scale (18).

Digital health interventions (DHIs) enable personalised care and empowerment through self-management of a long-term condition, which can be used at any time, and may offer more cost-effective health care delivery (19). DHIs have been shown to be effective at achieving successful remote monitoring and management of COPD amongst older populations (20), suggesting that DHIs may be a feasible method of reaching remote communities. Studies show that digital approaches for lifestyle modifications in other long-term conditions such as Type 2 diabetes, offer effective and scalable options when face-to face or in-person programmes are not accessible or feasible, especially when offered with health coaching (21, 22).

Health Coaching was originally described as the practice of health education and health promotion within a coaching context, to enhance the wellbeing of individuals and to facilitate achievement of health-related goals (23). More recently, HC has been defined as helping people gain and use the knowledge, skills, and confidence to become active participants in their care so that they can reach

For peer review only - http://bmjopen.bmj.com/site/about/guidelines.xhtml

their self-identified health and wellbeing goals (24). In the last decade, HC has become increasingly embedded within Integrated Care Services. However, the evidence base demonstrating the effectiveness of HC in the UK is limited. For example, a systematic review and meta-analysis assessing the impact of HC on Type 2 diabetes found that HC was effective in achieving optimal glycaemic levels via self-management strategies (25). In the UK, health coaches working within primary care can be an established clinical team member (e.g., registered nurses, physician associates, physiotherapists, paramedics, occupational therapists, and psychologists) or can be unlicensed health workers (26). Furthermore, research is emerging to show that HC may offer a promising approach to support effective lifestyle interventions for managing chronic conditions such as asthma, diabetes, cardiovascular disease, and mental illness on an international stage (27).

Current Integrated Care Systems in the UK have also implemented both DHIs via mHealth and HC to improve self-management of their long-term conditions (LTC), amongst older people living with COPD, diabetes, and cardiovascular disease (28). However, there remains a distinct lack of evidence which demonstrates the effectiveness of HC for older adults who have at least two chronic conditions (multimorbidity), which is thought to affect up to 50% of older people (aged over 65 years) (29, 30). Further, the impact of HC for older adults with a diagnosis of frailty, is unknown, making a single disease focused model of healthcare unsuitable to provide effective management (31). Therefore, targeted behaviour change interventions using DHIs such as mHealth in conjunction with HC has potential to offer a proactive, and wide-reaching approach to prevent frailty decline alongside the management of other diagnosed co-morbidities and improve health and wellbeing outcomes.

This study aims to address this gap by co-designing and implementing a multicomponent intervention for people living with mild-moderate frailty. The DIALOR intervention will include two key components (Figure 1);

1) Digital health coaching
    - This will include digital health coaching sessions with a qualified health coach. An online platform (Zoom/Accurx/Microsoft Teams) will be used to deliver the digital HC intervention.
2) Mobile health platform
    - A suite of web-based platforms offering tailored clinical advice and support and self-management resources for management of chronic obstructive pulmonary disease (COPD), Asthma, cardiovascular disease (CVD) and type 2 diabetes (details of the online platform can be accessed via https://mymhealth.com/). The platforms can be accessed by any electronic device (e.g., smartphone, tablet, or television) that has an active internet connection.

The primary objectives of this study are to:

- Explore barriers and facilitators of the digital intervention from the perspectives of health coaches and healthcare professionals (HCPs) and determine the essential and desirable features of the intervention (wants and needs; preferred outcomes) to identify what functions will be most useful for the target population.

For peer review only - http://bmjopen.bmj.com/site/about/guidelines.xhtml

- Explore how HC can provide support for the management of mild-moderate frailty and long-term conditions from the perspectives of older people with mild-moderate frailty, carers, HCPs, and health coaches.
- Determine the acceptability and feasibility of the intervention for older adults with mild/moderate frailty and another long-term condition referred to access health coaching services and willing to engage with the mobile health platform.

The secondary objectives of this study are:

- Explore the factors which inhibit or facilitate implementation including contextual factors, recruitment processes, resources needed, potential mechanisms and pathways to impact
- Explore the acceptability and suitability of outcomes measures (to inform sample size estimation and determine the primary outcome measure) for a future RCT (Randomised Control Trial) trial of the intervention (should it be feasible)
- Measure key domains for secondary outcome measures including participants who are able to self-report data for assessments

Figure 1: Overview of the DIALOR intervention and its functionalities

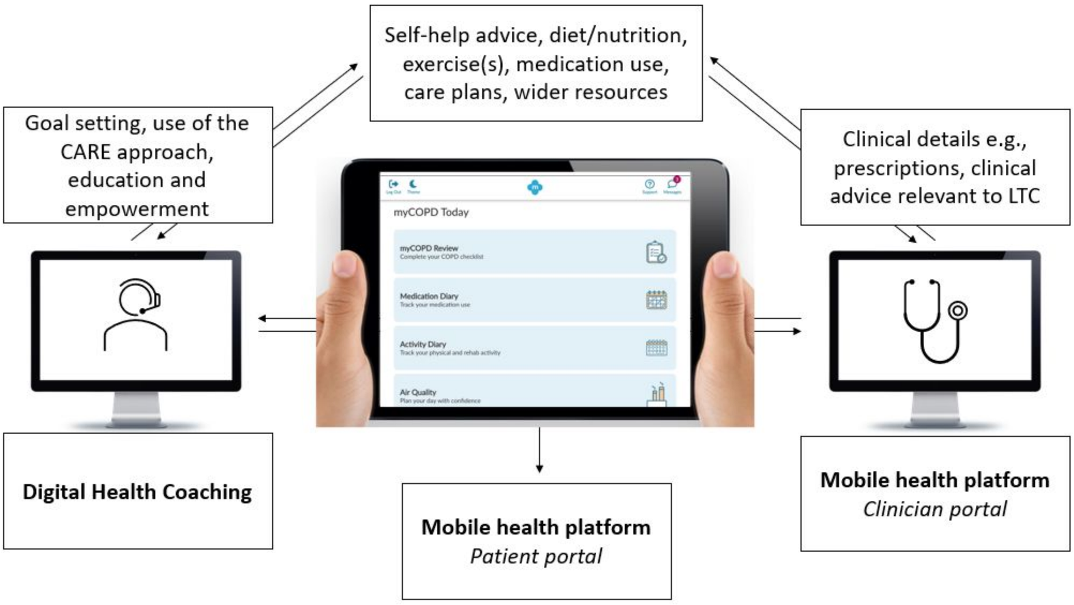

Methods and Analysis

Intervention Development

The aim is to develop an intervention using guiding principles (32), behavioural analysis (33) and the co-design of a logic model (Figure 2). A systematic review and narrative synthesis will inform the development of guiding principles to determine what our intervention needs to include to meet user needs and best practice guidelines as well as the format and amount of contact with the health coach. Behavioural analysis (33) will be using evidence from the review as well as consultation with two Stakeholder Engagement Groups (SEG) including HCPs, health coaches, commissioners of service and patient and public involvement (PPI) advisors as well as a separate PPI group with lay advisors to identify barriers in each domain - activity/mobility, nutrition, social engagement, and psychological well-being. Intervention components addressing each barrier will be coded using Behaviour Change Taxonomy (BCT) (34) and Normalisation Process Theory (NPT) which is the process through which complex, new ways of working become embedded into standard practice (35), to provide a clear description of the intervention and factors which may influence its implementation. We will then

For peer review only - http://bmjopen.bmj.com/site/about/guidelines.xhtml

convene another SEG meeting to discuss and adapt the proposed logic model. The intervention will then be evaluated with key stakeholders.

To identify challenges/enabling factors for delivery/implementation and intervention modifications needed to maximise engagement and behaviour change, we will conduct individual interviews with people with mild to moderate frailty (and their carers where appropriate) (n=20) and focus groups (n=4) with HCPs and health coaches (n=20). These will be conducted face-to-face, and audio recorded using dictaphones, otherwise they will be conducted and recorded through Microsoft Teams or Zoom. Final in-house testing of the intervention will be undertaken before the commencement of study. We will use the TIDieR checklist (36) for intervention description which will report relevant outcomes to inform further planning for a future trial. We do not foresee any adverse events occurring which are generally related to the intervention. However, adverse events may occur, as with most clinical trials, such as hospitalisation or death which may be directly related to participants pre-existing medical conditions. This may be normal amongst the populations which we are studying.

Study Design

This feasibility study is a non-randomised, single group, pretest/post-test study, using qualitative and quantitative methods. This protocol was developed in accordance with the Standard Protocol Items: Recommendations for Intervention Trials (SPIRIT) reporting guidelines (37).

This will be a mixed methods prospective cohort study design conducted within primary care settings in Southwest England. Firstly, online group training of the intervention will be provided for health coaches by the research team, with top-up training and support as necessary throughout . A training manual will also incorporate frailty awareness, and the CARE (Congratulate, Ask, Reassure, Encourage) model approach (38, 39). Intervention delivery will be tailored to each patient, with goals and strategies to help achieve them developed in conjunction with the health coach.

Study setting

The study will work with GP practices in Primary Care Networks (PCN) in Southwest England to identify patients already using the mobile health platform. GP practices will be eligible to take part if they are currently referring older people with mild to moderate frailty to access HC/social prescribing services and willing to test new digital approaches.

Participant recruitment

Participant recruitment will be conducted within primary care services, at individual participating GP surgeries. Up to 50 older people with mild-moderate frailty will be recruited in line with previous sample size recommendations for feasibility studies (40). Eligibility criteria for older adults with mild to moderate frailty to participate in the study are outlined in Table 1. The clinical care team from participating GP surgeries will identify eligible participants through existing medical records.

Three strategies will be utilised to recruit patients i) via text message invitation to eligible patients which provides a link to a website with further information about the study, participant information sheet and contact information for the research team. Potential participants would be followed up by the research team to provide an opportunity to answer questions, confirm eligibility and obtain informed consent to participate, ii) by a member of the research team approaching eligible participants at the GP surgery, signposted by the clinical team and iii) via leaflets provided to eligible patients by the clinical team.

Table 1: Inclusion and exclusion criteria

Inclusion criteria
• Aged ≥ 65 years

For peer review only - http://bmjopen.bmj.com/site/about/guidelines.xhtml

• Scoring as mild or moderately frail using the Electronic Frailty Index<br>• Diagnosis of 1 or more long-term conditions including COPD, Type 2 diabetes, heart disease or asthma<br>• Living in their own homes<br>• Capacity to consent to participate<br>• Able to communicate fluently enough in English to take part in the study<br>• Able to recall their experiences sufficiently to engage with a health coach<br>• Self-reported ability to use digital technology independently or with support
Exclusion criteria
• Nursing home resident or on active waiting list for a place<br>• Individuals who have a terminal illness and are being managed for end-of-life needs<br>• Lacking capacity to consent (e.g., advanced dementia)

Figure 2: Proposed logic model

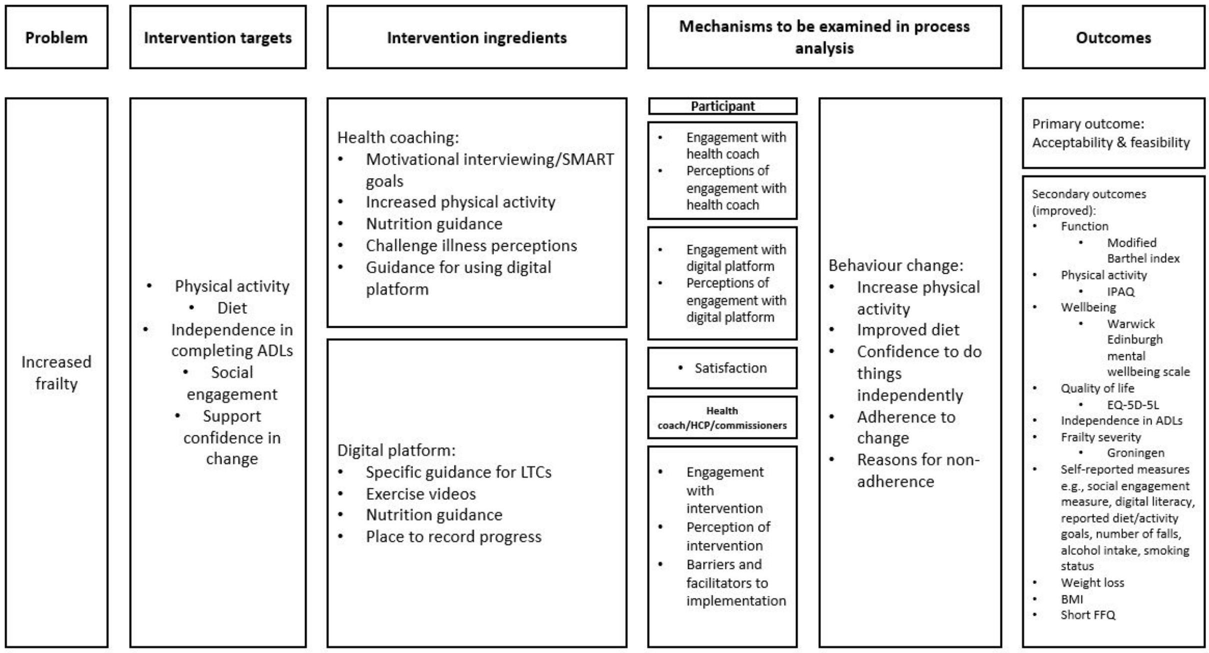

Figure 3: Process flow of study design

For peer review only - http://bmjopen.bmj.com/site/about/guidelines.xhtml

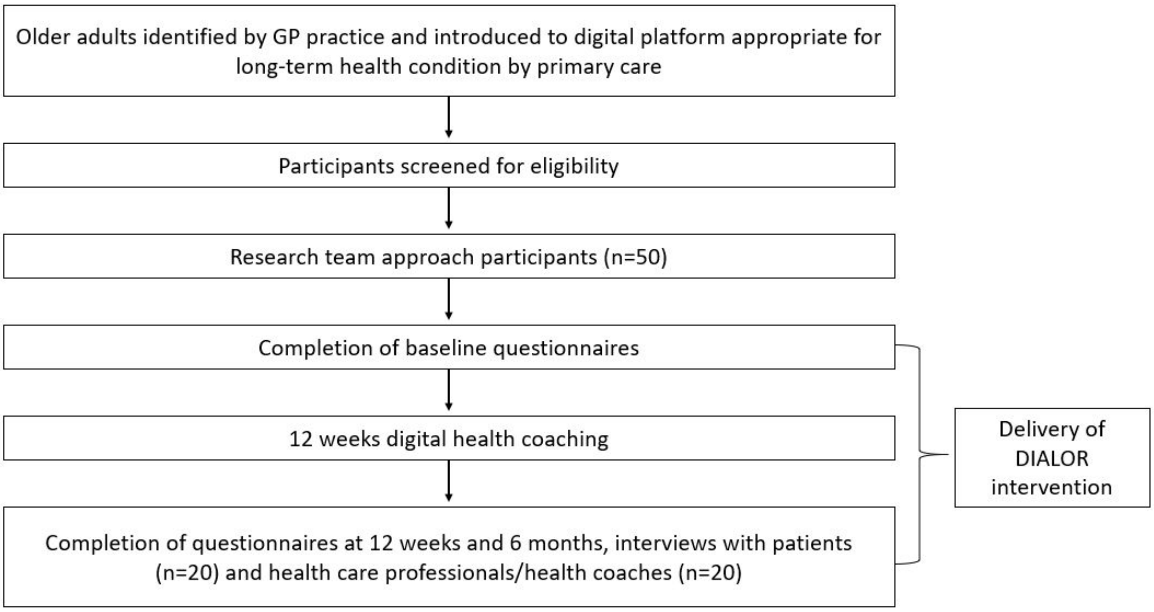

Digital Support for patients

Patients with low digital literacy will be supported by the research team to access the digital HC and mobile health platforms. They will be offered a training manual, developed by the research team, and co-produced alongside the SEG and PPI group alongside digital support leaflets and guidance on keeping safe online. To reduce the risk of digital inequity, internet enabled tablets may be provided to individuals who lack a device, for the length of the study. Internet and data packages (via local council schemes) will be provided if the participant lacks access to an established internet connection. Digital support will be offered throughout the study via local council services (volunteer digital upskilling clinics embedded within public spaces e.g., GP surgeries and council libraries) and the research team.

Outcome measures

Primary outcomes

The primary outcome measures are feasibility and acceptability of the DIALOR intervention. Feasibility will be determined from the number of health coaches trained, number of participants recruited retention and adherence to the intervention as well as any adverse events. To determine the acceptability of the intervention and to explore barriers and enablers to the implementation of the intervention, semi-structured interviews will be conducted with patients with mild-moderate frailty (and their carers where appropriate) (n= 20), HCPs and health coaches (n= 20) who participated in the study.

The interviews will consist of open-ended questions to explore participant views of the intervention, barriers, and facilitators to inform the design of further studies. To understand the factors influencing implementation, the interview schedule will be underpinned by normalisation process theory (NPT) (41). NPT is an implementation theory providing a framework to identify and explain important elements of the implementation process. As such it informs an understanding of the social processes through which new or practice can be implemented, embedded, and integrated into care settings.

For peer review only - http://bmjopen.bmj.com/site/about/guidelines.xhtml

Secondary outcomes

The secondary outcomes will include the measurement of functioning, quality of life, physical activity levels, frailty severity, diet quality, and engagement using validated outcome measures. Physical functioning, a key outcome in frailty trials (42), will be measured using the Modified Barthel Index (MBI) (43) and Lawton-Brody Instrumental Activities of Daily Living (IADL) (44). The MBI will measure older adults' functional ability using 10 items, with a higher score reflecting higher ability to function independently. The MBI has been found to be of reasonable reliability when used amongst older adults (45). The IADL will measure more complex activities of daily living using 8 key domains. The International Physical Activity Questionnaire short form (IPAQ-SF) will measure older adults activity levels in the last 7 days (46). The evidence supporting the IPAQ-SF as an indicator of relative or absolute physical activity is weak (47). However, it is a validated generalised outcome measure for older adult populations (65 years or over) (48).

Quality of life is a core outcome in frailty trials (49) and will be measured using the EuroQol-5dimsensions-5levels (EQ-5D-5L) questionnaire which includes 5 health areas (mobility, self-care, usual activities, pain/discomfort, and anxiety/depression). These 5 health areas are assessed with 5 main response levels (no problems, slight, moderate, severe, or unable), along with a visual analogue scale (50).

Frailty severity will be measured using the Groningen Frailty Indicator (GFI) (51). The GFI is a multidimensional screening instrument consisting of 15 self-report items. It is a feasible, and widely used screening instrument for identifying frail older adults (52, 53). Higher GFI scores indicate higher frailty levels and an increased need for integrated care (54).

The Short Form Food Frequency Questionnaire (SFFFQ) will measure dietary quality. The SFFQ asks about foods which respondents might have consumed, on average, over a set time period (3 months) and is found to be an effective instrument of assessing diet quality in large scale studies with UK adult populations (55).

Social engagement will be measured using the Lubben Social Network Scale (LSNS-6) and mental wellbeing will be measured using the Warwick-Edinburgh Mental Well-Being Scale (WEMWBS)

Self-reported indicators such as height, weight, BMI (Body Mass Index), number of falls (per year), goal attainment, alcohol intake and smoking habits (if applicable) will be measured at baseline (pre-intervention), throughout the study during digital HC intervention and at 6 months (post-intervention).

Data will collected from the mobile health platform which will be recorded automatically by inbuilt tracking software including usage patterns from number of logins, and length of time that older people and their carers use the platform. This data will be collected at 6 months for each patient participant (post-intervention).

Data Analysis

Quantitative data analysis

Data will be entered into a secured database for analyses. Statistical analyses will be conducted using statistical software SPSS (V.28.0). Descriptive statistics: median (IQR); mean (SD); number (%) and confidence interval – will be used to analyse the number of volunteers recruited and retained, as well as participants adherence to the intervention and pre-post intervention outcome measures to assess the feasibility of delivering the intervention. Pre and post analyses at 6 months, will be conducted to determine if the intervention had an impact on the lifestyle management of frailty measured by functioning (MBI, IADL), social engagement (LSNS-6), mental wellbeing (WEMWBS)

For peer review only - http://bmjopen.bmj.com/site/about/guidelines.xhtml

quality of life (EQ-5D-5L), physical activity (IPAQ-SF), frailty indicator (GFI), diet quality (SFFFQ) and self-reported indicators.

Qualitative data analysis

Data collected from all the interviews and focus groups will be transcribed verbatim and analysed using thematic analysis (TA). TA is a commonly used method for identifying, analysing, and reporting patterns or themes within data (56). There are six key phases: Phase 1 – familiarisation with the data, Phase 2 – initial code generation, Phase 3 – searching for and generating themes, Phase 4 – reviewing themes, Phase 5 – defining and naming themes and Phase 6 – producing the report. Analysis of qualitative data will be conducted using Microsoft word, and or alternative software such as NVivo (V.12). RC will analyse codes using NPT (35) and the Behaviour Change Technique Taxonomy (34) to focus on the social processes shaping the implementation process from making sense of the intervention to engaging in it (individually and collectively) and embedding this (normalising) in everyday practice (or people's everyday lives) and to identify the facilitators and barriers present throughout the implementation process. Themes will be developed, from the codes, to reflect the lived experience and views of patient participants, health coaches, and HCPs regarding the digital HC and mobile health platform intervention. MS will code 25% of interviews and focus groups separately to develop, discuss, and agree themes with RC. Finally, members of the core research team (ES, MB, CC, JM) will be consulted to develop, discuss, and agree themes via an iterative process.

Patient and Public Involvement

Public/patient involvement and engagement (PPI) has been and will continue to be part of all stages of the research from developing the proposal to providing feedback on preliminary findings, to dissemination and impact, and input for tasks such as finalising interview/focus group topic guides. For the development of this programme of research, we consulted with seven older adults and family carers who helped inform the proposal. They thought that this was an important topic and liked the HC approach to support digital engagement. We have convened a PPI advisory group (6-10 participants, aged 60+ years, meeting 3 to 4 times throughout the duration of the project). A PPI representative as a co-researcher leads the PPI group and attends the project advisory group. The group represents the views of people from different socio-economic backgrounds including those who do and do not have access to various online and digital technologies.

PPI meetings will be face to face and advisors will be offered re-imbursement aligning with National Institution for Health and Care Research (NIHR) payment guidance. Advisors will have the opportunity to contribute via email and phone between meetings. Advisors will be sent materials prior to meetings and able to discuss matters arising from the meeting afterwards. Advisors will be provided with meeting summaries and feedback on how their involvement informed the research project. A PPI plan will be developed and shaped by PPI and results/activities will be shared with the project advisory group. Monitoring and reporting will use the GRIPP checklist (57).

Ethics and dissemination

The DIALOR project is sponsored by Bournemouth University. Ethical approval has been granted by the Southeast Scotland Research Ethics Committee 02 (reference: 22/SS/0064). Data collected during the study will be pseudonymised by a member of the research team (RC) to ensure removal of identifying information from research data prior to data analysis. Data will be stored on

For peer review only - http://bmjopen.bmj.com/site/about/guidelines.xhtml

Bournemouth University servers, and identification data will be stored in encrypted files in a separate, secure location. Data transfer will be undertaken through secure, encrypted emails or Bournemouth University's secure file transfer system (BU Transfer). We will publish results in a peer-reviewed open access journals. A lay summary of the final study report will be accessible on the project website: (https://www.bournemouth.ac.uk/research/projects/dialor-digital-coaching-frailty). We will also seek to present our findings at conferences, workshops for patients, write lay articles for dissemination to the public, and share the findings through our networks across health and social care.

Status

The trial commenced September 2023 and is expected to be completed by September 2024.

Acknowledgements

The authors thank study participants and patient advisers for their input. The authors wish to thank GP director Simone Yule for helping to implement the study within local primary care services. The authors would like to thank colleagues from Dorset Help & Care charity and my mhealth for helping to implement the study intervention and imbed this seamlessly within primary care services.

Author Contributions

The study concept and design was conceived by JM. MS, RC, ES, CC, and MB assisted in refining the study questionnaires and study design. MS, RC, and MAP will be responsible for data collection. Analyses will be conducted by MS, RC, ES, DMW, CS, KB, CC, MB, JM, and MAP. MS prepared the first draft of the manuscript. All authors critically revised the manuscript and approved the submitted version. The corresponding author attests that all listed authors meet authorship criteria and that no others meeting the criteria have been omitted.

Funding

This research is supported by NIHR Applied Research Collaborative Wessex, grant number ARC001.

Disclaimer

Provenance and peer review not commissioned; externally peer reviewed.

Competing interests

None declared.

Copyright/license

The Corresponding Author has the right to grant on behalf of all authors and does grant on behalf of all authors, a worldwide licence to the Publishers and its licensees in perpetuity, in all forms, formats and media (whether known now or created in the future), to i) publish, reproduce, distribute, display and store the Contribution, ii) translate the Contribution into other languages, create adaptations, reprints, include within collections and create summaries, extracts and/or, abstracts of the Contribution, iii) create any other derivative work(s) based on the Contribution, iv) to exploit all subsidiary rights in the Contribution, v) the inclusion of electronic links from the Contribution to

For peer review only - http://bmjopen.bmj.com/site/about/guidelines.xhtml

third party material where-ever it may be located; and, vi) licence any third party to do any or all of the above.

For peer review only

For peer review only - http://bmjopen.bmj.com/site/about/guidelines.xhtml

References

1. Rockwood, K., Song, X., MacKnight, c., Bergman, H., Hogan, D., McDowell, I., Mitnitski, A., 2005. A global clinical measure of fitness and frailty in elderly people, *Canadian Medical Association Journal*, 173 (5), 489-495.
2. Clegg, A., Young, J., Iliffe, S., Rikkert, M., Rockwood, K., 2013. Frailty in elderly people, *Lancet*, 381 (9868), 1-25.
3. Langlois, F., Vu, T., Chasse, K., Dupuis, G., Kergoat, M J., Bherer, L., 2012. Benefits of physical exercise training on cognition and quality of life in frail older adults, The *Journals of Gerontology, Series B, Psychological Sciences and Social Sciences,* 68 (3), 400-404.
4. Collard, R., Comijs, H., Naarding, P., Penninx, B., Milaneschi, Y., Ferruci, L., Voshaar, R., 2015. Frailty as a Predictor of the Incidence and Course of Depressed Mood, *Journal of the American Medical Directors Association*, 16 (6), 509-514.
5. Robertson, D., Savva, G., Kenny, R., 2013. Frailty and cognitive impairment – A review of the causal mechanisms, *Ageing Research Reviews*, 12 (4), 840-851.
6. O'Caoimh, R., Sezgin, D., O'Donovan, M,R., Molloy, D,W., Clegg, A., Rockwood, K., 2021. Prevalence of frailty in 62 countries across the world: a systematic review and meta-analysis of population-level studies, Age Ageing, 50 (1), 96–104.
7. Gale, C., Cooper, C., Deary I., Sayer A., 2014. Psychological well-being and incident frailty in men and women: the English Longitudinal Study of Ageing, *Psychological Medicine*, 44 (4), 697-706.
8. Collard, R., Boter, H., Schoevers, R., Voshaar, R., 2012. Prevalence of Frailty in Community-Dwelling Older Persons: A Systematic Review, *Journal of the American Geriatrics Society*, 60 (8), 1487-1492.
9. Hussien, H., Nastasa, A., Apetrii, M., Nistor, I., Petrovic, M., Covic, A., 2021. Different aspects of frailty and COVID-19: points to consider in the current pandemic and future ones, *BMC Geriatrics*, 21 (389), 1-11.
10. Steinman, M., Perry, L., Perissinotto, C., 2020. Meeting the Care Needs of Older Adults Isolate at Home During the COVID-19 Pandemic, *JMA Internal Medicine*, 180 (6), 819-820.
11. Ng, T., Feng, L., Nyunt, M., Feng, L., Niti, M., Tan, B., Chan, G., Khoo, S., Chan, S., Yap, P., Yap, K., 2015. Nutritional, Physical, Cognitive, and Combination Interventions and Frailty Reversal Among Older Adults: A Randomized Controlled Trial, *The American Journal of Medicine*, 128 (11), 1225-1236.
12. Gill, T., Gahbauer, E., Allore, H., Han L., 2006. Transitions between frailty states among community-living older persons, *Archives of Internal Medicine*, 166 (4), 418-423.
13. Rockwood, K., Theou, O., Mitnitski, A., 2015. What are frailty instruments for? *Age and Ageing*, 44, 545-547.
14. Cameron, I., Fairhall, N., John, B., Lockwood, K., Monaghan, N., Sherrington, C., 2017. A multifactorial interdisciplinary intervention in pre-frail older people: randomised trial, *Innov Aging*, 1 (196)
15. Walters K, Frost R, Kharicha K, Avgerinou C, Gardner B, Ricciardi, F., 2017. Home-based health promotion for older people with mild frailty: the Home Health intervention development and feasibility RCT, *Health Technol Assess*, 21 (73), 1–128.

For peer review only - http://bmjopen.bmj.com/site/about/guidelines.xhtml

16. Serra-Prat, M., Sist, X., Domenich, R., Jurado, L., Saiz, A., Roces, A., 2017. Effectiveness of an intervention to prevent frailty in pre-frail community-dwelling older people consulting in primary care: a randomised controlled trial, Age Ageing, 46 (3), 401–7.
17. Yu, R., Tong, C., Ho, F., Woo, J., 2020. Effects of a Multicomponent Frailty Prevention Program in Prefrail Community-Dwelling Older Persons: A Randomized Controlled Trial. J Am Med Dir Assoc, 21 (2), e1- e10.
18. Chen, Z., Ding, Z., Chen, C., Sun, Y., Jiang, Y., Liu, F., Wang, S., 2021. Effectiveness of comprehensive geriatric assessment intervention on quality of life, caregiver burden and length of hospital stay: a systematic review and meta-analysis of randomised controlled trials, *BMC Geriatrics*, 21(1), 377.
19. Gomes, M., Murray, E., Raftery, J., 2022. Economic Evaluation of Digital Health Interventions: Methodological Issues and Recommendations for Practice, *PharmacoEconomics*, 40, 367-378.
20. Velardo, C., Shah, S., Gibson, O., Clifford, G., Heneghan, C., Rutter, H., Farmer, A., Tarassenko, L., 2017. Digital health system for personalised COPD long-term management, *BMC Medical Informatics and Decision Making*, 17 (19), 1-13.
21. Smith, A., Crippa, A., Woodcock, J., Brage, S., 2016. Physical activity and incident type 2 diabetes mellitus: a systematic review and dose-response meta-analysis of prospective cohort studies, *diabetologia*, 59 (12), 2527-2545.
22. Castro Sweet, C., Chiguluri, V., Gumpina, R., Abbott, P., Madero, E., Payne, M., Happe, L., Matanich, R., Renda, A., Prewitt, T., 2018. Outcomes of a Digital Health Program With Human Coaching for Diabetes Risk Reduction in a Medicare Population, *Journal of Aging and Health*, 30 (5), 692-710.
23. Palmer, S., Cooper, C., Thomas, K., 2003. Creating a Balance: Managing Stress. London: British Library.
24. NHS England 2019. Universal Personalised Care: Implementing the Comprehensive Model.
25. Pirbaglou, M., Katz, J., Motamed, M., Pludwinski, S., Walker, K., Ritvo, P., 2018. Personal Health Coaching as a Type 2 Diabetes Mellitus Self-Management Strategy: A Systematic Review and Meta-Analysis of Randomized Controlled Trials, *American Journal of Health Promotion*, 32 (7), 1613-1626.
26. Gastala, N., Wingrove, P., Gaglioti, A., Liaw, W., Bazemore, A., 2018. The Growing Trend of Health Coaches in Team Based Primary Care Training, *Family medicine*, 50 (7), 526-530.
27. Yang, J., Bauer, B., Lindeen, S., Perlman, A., Dabrh, A., Boehmer, K., Salinas, M., Cutshall, S., 2020. Current trends in health coaching for chronic conditions., *Open Medicine*, 99 (30), 1-6.
28. McShane, M. and Kirkham, K. (2020). Making it personal – population health management and the NHS", *Journal of Integrated Care*, 28 (3), 243-252.
29. Barnett, K., Mercer, SW., Norbury, M., Watt, G., Wyke, S., Guthrie, B., 2012. Epidemiology of multimorbidity and implications for health care, research, and medical education: a cross-sectional study. *Lancet 2012*; 380 (9836), 37–43.
30. Salive, ME., 2013. Multimorbidity in older adults. *Epidemiol Rev*, 35, 75–83.
31. Kingston, A., Robinson, L., Booth, H., Knapp, M., Jagger, C., 2018. Projections of multi-morbidity in the older population in England to 2035: estimates from the Population Ageing and Care Simulation (PACSim) model, *Age and Ageing*, 47 (3), 374-380.

For peer review only - http://bmjopen.bmj.com/site/about/guidelines.xhtml

32. Yardley, L., Morrison, L., Bradbury, K., Muller, I., 2015. The person-based approach to intervention development: application to digital health-related behavior change interventions, *Journal of medical Internet research*, 17 (1), 1-15.
33. Band, R., Bradbury, K., Morton, K., May, C., Michie, S., Mair, F., Murray, E., McManus R., Little, P., Yardley, L., 2017. Intervention planning for a digital intervention for self-management of hypertension: a theory-, evidence- and person-based approach, *Implementation Science*, 12 (25), 1-13.
34. Michie, S., Richardson, M., Johnston, M., Abraham, C., Francis, J., Hardeman, W., Eccles, M., Cane, J., Wood, C., 2013. The behaviour change taxonomy (v1) of 93 hierarchically clustered techniques: building an international consensus for the reporting of behaviour change interventions, *Annals of Behavioural Medicine*, 46 (1), 81-95.
35. May, C., Cummings, A., Girling, M., Bracher, M., Mair, F., May, C., Murrray, E., Myall, M., Rapley, T., Finch, T., 2018. Using Normalization Process Theory in feasibility studies and process evaluations of complex healthcare interventions: a systematic review, *Implementation Science*, 13 (80), 1-27.
36. Hoffman, T., Glasziou, P., Milne, R., Moher, D., Barbour, V., Johnston, M., Lamb, S., Dixon-Woods, M., Wyatt, J., 2014. Better reporting of interventions: a template for intervention description and replication (TIDieR) checklist and guide, *BMJ*, 348, 1-12.
37. Chan, A, W., Tetzlaff, J., Gøtzsche, P., Altman, D., Mann, H., Berlin, J., Dickersin, K., *Hróbjartsson, A., Schulz, K., Parulekar, W., Krleža-Jerić, K., Laupacis, A., Moher, D., 2013.* SPIRIT 2013 statement: defining standard protocol items for clinical trials, *Annals of Internal Medicine*, 158 (3), 200-207.
38. Bradbury, K., Morton, K., Band, R., May, C., McManus, R., Little, P., Yardley, L., 2017. Understanding how primary care practitioners perceive an online intervention for the management of hypertension, *BMC Medical Informatics and Decision* Making, 17 (5), 1-13.
39. Smith, E., Bradbury, K., Scott, L., Steele, M., Little, P., Yardley, L., 2017. Providing online weight management in primary care: a mixed methods process evaluation of healthcare practitioners' experiences of using and supporting patients using POWeR+, *Implementation Science*, 12 (69), 1-13.
40. Billingham, S., Whitehead, A., Julious, S., 2013. An audit of sample sizes for pilot and feasibility trials being undertaken in the United Kingdom registered in the United Kingdom Clinical Research Network database, *BMC Medical Research and Methodology*, 13 (104), 1-6.
41. May, C., Mair, F., Finch, T., MacFarlane, A., Dowrick, C., Treweek, S., Rapley, T., Ballini, L., Ong, B., Rogers, A., Murray, E., Elwyn, G., Légaré, f., Gunn, J., Montori, V., Development of a theory of implementation and integration: Normalization Process Theory, *Implementation Science*, 4 (29), 1-9.
42. Tappenden, P., Campbell, F., Rawdin, A., Wong, R., Kalita, N., 2012. The clinical effectiveness and cost-effectiveness of home-based, nurse-led health promotion for older people: a systematic review, *Health Technology Assessment*, 16 (20), 1-72.
43. Wade, DT., and Collin, C., 1988. The Barthel ADL Index: a standard measure of physical disability, *International Disability Study*, 10, 64-67
44. Lawton, M.P., and Brody, E.M, 1969. Assessment of older people: Self-maintaining and instrumental activities of daily living. The Gerontologist, 9 (3), 179-186.

For peer review only - http://bmjopen.bmj.com/site/about/guidelines.xhtml

45. Hopman-Rock, M., Hirtum, H., Vreede, P., Freiberger, E., 2019. Activities of daily living in older community-dwelling persons: a systematic review of psychometric properties of instruments, *Aging Clinical and Experimental Research*, 31, 917-925.
46. Hagstromer, M., Oja, P., Sjostrom, M., 2006. The international physical activity questionnaire (IPAQ): a study of concurrent and construct validity. *Public Health Nutrition*, 9 (6), 755-762.
47. Lee, P., Macfarlane, D., Lam, T., Stewart, S., 2011. Validity of the international physical activity questionnaire short form (IPAQ-SF): A systematic review. *International Journal of Behavioural Nutrition and Physical Activity*, 8 (115), 1-11.
48. Hurtig-Wennlöf, A., Hagströmer, M., Olsson, LA., 2010. The International Physical Activity Questionnaire modified for the elderly: aspects of validity and feasibility. *Public Health Nutr*, 13, 1847–54.
49. Prorok, J., Williamson, P., Shea, B., Rolfson, D., Manas, L., Cesari, M., Kim, P., Muscedere, J., 2022. An international Delphi consensus process to determine a common data element and core outcome set for frailty: FOCUS (The Frailty Outcomes Consensus Project), *BMC Geriatrics*, 22 (284), 1-9.
50. The EuroQoL Group. EuroQol--a new facility for the measurement of health-related quality of life. Health Policy 1990;16:199-208.
51. Steverink, N., 2001. Measuring frailty: developing and testing the GFI (Groningen frailty Indicator). *The Gerontologist*, 41, 236.
52. Metzelthin S, Daniëls R, Van Rossum E, De Witte L, van den Heuvel W, Kempen G., 2010. The psychometric properties of three self-report screening instruments for identifying frail older people in the community. *BMC Publ Health*, 10 (1), 176–183.
53. Peters LL, Boter H, Buskens E, Slaets JPJ., 2012. Measurement properties of the groningen frailty indicator in home-dwelling and institutionalized elderly people, *J Am Med Dir Assoc*, 13, 546–551.
54. Slaets, J,P., 2006. Vulnerability in the elderly: frailty. *Med Clin North Am*, 90 (4), 593–601.
55. Cleghorn, C., Harrison, R., Ransley, J., Wilikinson, S., Thomas, J., Cade, J., 2016. Can a dietary quality score derived from a short-for FFQ assess dietary quality in UK adult population surveys? *Public Health Nutrition*, 19 (16), 2915-2923.
56. Braun, V., Clarke, V., 2014. What can " thematic analysis " offer health and wellbeing researchers? *Int J Qual Stud Health Well-Being*, 9 (26152), 1-2.
57. Staniszewska, S., Brett, J., Simera, I., Seers, K., Mockford, C., Goodlad, S., Altman, D., Moher, D., Barber, R., Denegri, S., Entwistle, A., Littlejohns, P., Morris, C., Suleman, R., Thomas, V., Tysall, C., 2017. GRIPP2 reporting checklists: tools to improve reporting of patient and public involvement in research, *BMJ*, 2017, 358, 1-7.

For peer review only - http://bmjopen.bmj.com/site/about/guidelines.xhtml

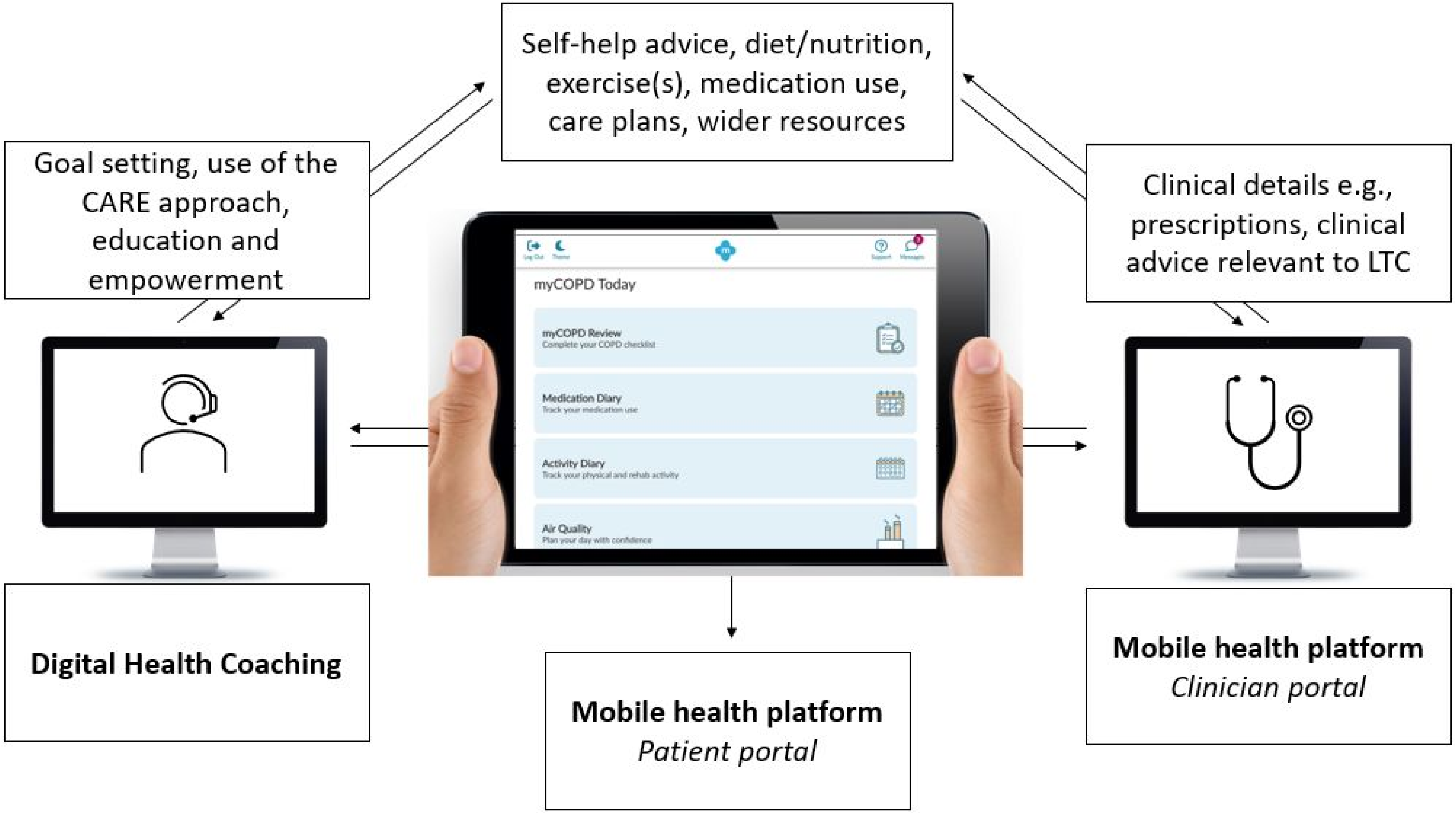

Figure 1: Overview of the DIALOR intervention and it’s functionalities

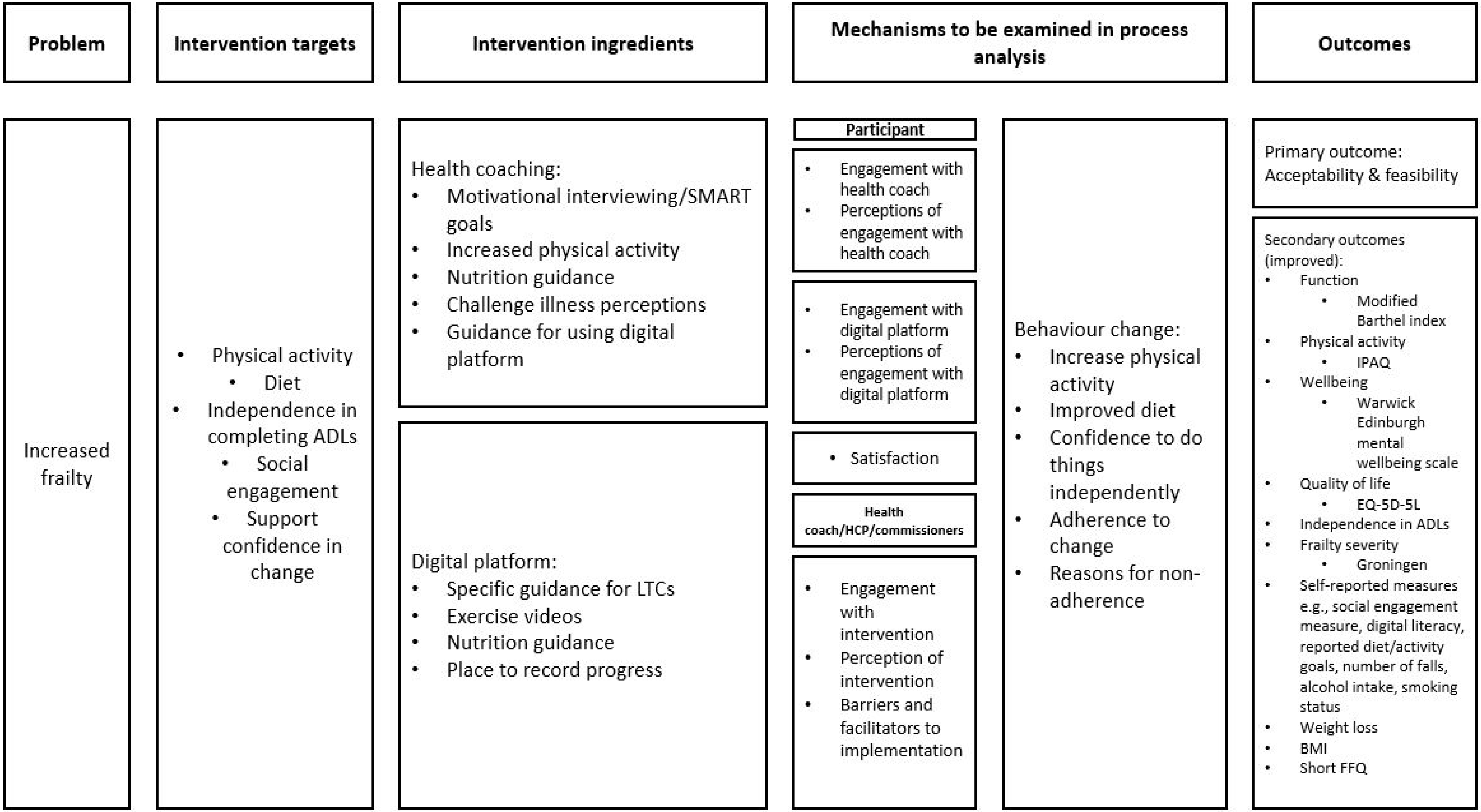

Figure 2: Proposed logic model

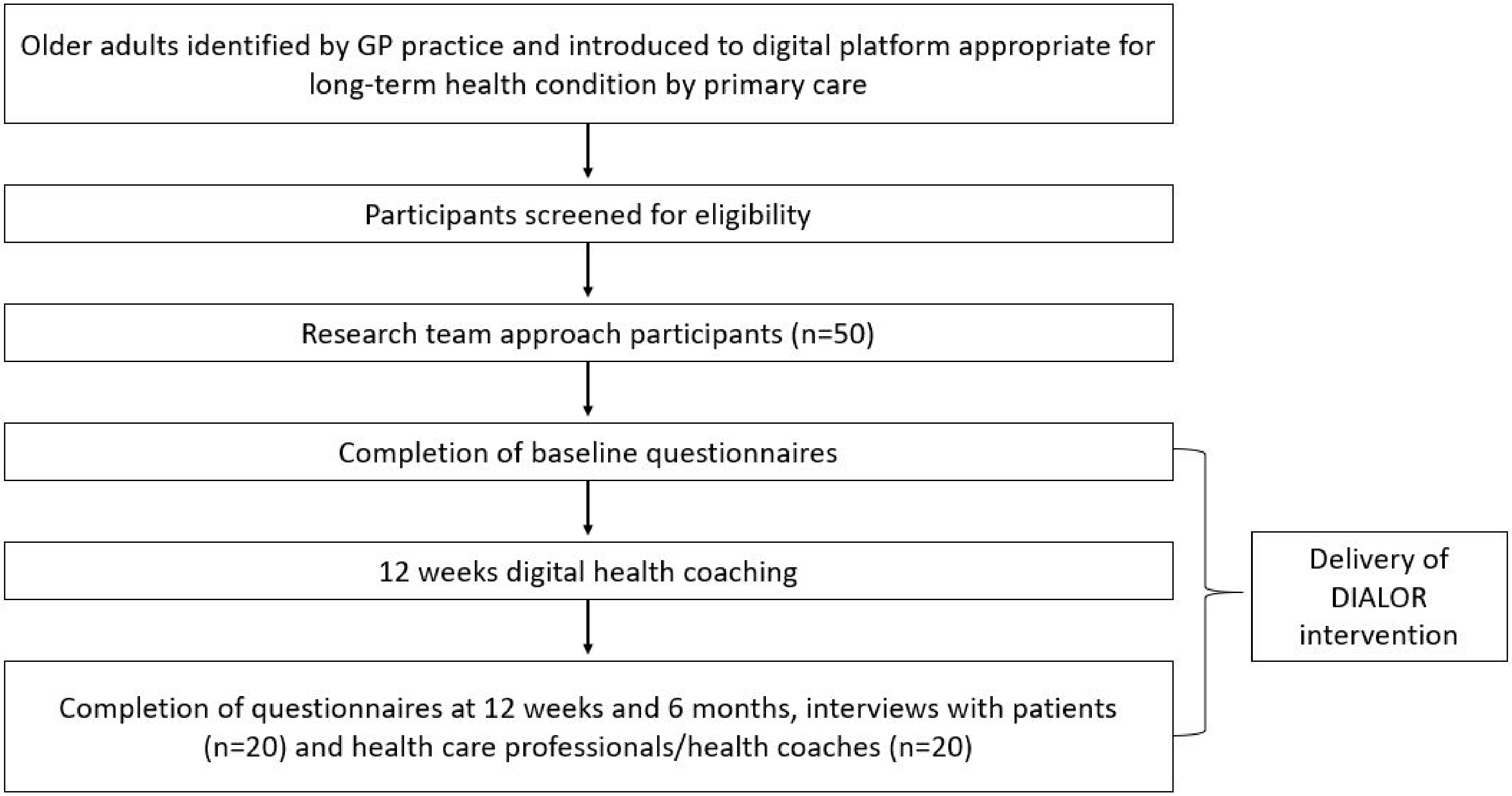

Figure 3: Process flow of study design

SPIRIT
STANDARD PROTOCOL ITEMS: RECOMMENDATIONS FOR INTERVENTIONAL TRIALS

SPIRIT 2013 Checklist: Recommended items to address in a clinical trial protocol and related documents*

Section/item	Item No	Description	Addressed on page number
**Administrative information**			
Title	1	Descriptive title identifying the study design, population, interventions, and, if applicable, trial acronym	1
Trial registration	2a	Trial identifier and registry name. If not yet registered, name of intended registry	1
	2b	All items from the World Health Organization Trial Registration Data Set	nil
Protocol version	3	Date and version identifier	1
Funding	4	Sources and types of financial, material, and other support	11
Roles and responsibilities	5a	Names, affiliations, and roles of protocol contributors	11
	5b	Name and contact information for the trial sponsor	11
	5c	Role of study sponsor and funders, if any, in study design; collection, management, analysis, and interpretation of data; writing of the report; and the decision to submit the report for publication, including whether they will have ultimate authority over any of these activities	11
	5d	Composition, roles, and responsibilities of the coordinating centre, steering committee, endpoint adjudication committee, data management team, and other individuals or groups overseeing the trial, if applicable (see Item 21a for data monitoring committee)	5,6,9,10

For peer review only

Introduction

Background and rationale	6a	Description of research question and justification for undertaking the trial, including summary of relevant studies (published and unpublished) examining benefits and harms for each intervention	3
	6b	Explanation for choice of comparators	
Objectives	7	Specific objectives or hypotheses	4,5
Trial design	8	Description of trial design including type of trial (eg, parallel group, crossover, factorial, single group), allocation ratio, and framework (eg, superiority, equivalence, noninferiority, exploratory)	6

Methods: Participants, interventions, and outcomes

Study setting	9	Description of study settings (eg, community clinic, academic hospital) and list of countries where data will be collected. Reference to where list of study sites can be obtained	6
Eligibility criteria	10	Inclusion and exclusion criteria for participants. If applicable, eligibility criteria for study centres and individuals who will perform the interventions (eg, surgeons, psychotherapists)	7
Interventions	11a	Interventions for each group with sufficient detail to allow replication, including how and when they will be administered	5,6
	11b	Criteria for discontinuing or modifying allocated interventions for a given trial participant (eg, drug dose change in response to harms, participant request, or improving/worsening disease)	n/a
	11c	Strategies to improve adherence to intervention protocols, and any procedures for monitoring adherence (eg, drug tablet return, laboratory tests)	n/a
	11d	Relevant concomitant care and interventions that are permitted or prohibited during the trial	n/a
Outcomes	12	Primary, secondary, and other outcomes, including the specific measurement variable (eg, systolic blood pressure), analysis metric (eg, change from baseline, final value, time to event), method of aggregation (eg, median, proportion), and time point for each outcome. Explanation of the clinical relevance of chosen efficacy and harm outcomes is strongly recommended	8,9
Participant timeline	13	Time schedule of enrolment, interventions (including any run-ins and washouts), assessments, and visits for participants. A schematic diagram is highly recommended (see Figure)	8

Sample size	14	Estimated number of participants needed to achieve study objectives and how it was determined, including clinical and statistical assumptions supporting any sample size calculations	6____________
Recruitment	15	Strategies for achieving adequate participant enrolment to reach target sample size	6____________

Methods: Assignment of interventions (for controlled trials)

Allocation:			
Sequence generation	16a	Method of generating the allocation sequence (eg, computer-generated random numbers), and list of any factors for stratification. To reduce predictability of a random sequence, details of any planned restriction (eg, blocking) should be provided in a separate document that is unavailable to those who enrol participants or assign interventions	n/a___________
Allocation concealment mechanism	16b	Mechanism of implementing the allocation sequence (eg, central telephone; sequentially numbered, opaque, sealed envelopes), describing any steps to conceal the sequence until interventions are assigned	n/a___________
Implementation	16c	Who will generate the allocation sequence, who will enrol participants, and who will assign participants to interventions	n/a___________
Blinding (masking)	17a	Who will be blinded after assignment to interventions (eg, trial participants, care providers, outcome assessors, data analysts), and how	n/a___________
	17b	If blinded, circumstances under which unblinding is permissible, and procedure for revealing a participant's allocated intervention during the trial	n/a___________

Methods: Data collection, management, and analysis

Data collection methods	18a	Plans for assessment and collection of outcome, baseline, and other trial data, including any related processes to promote data quality (eg, duplicate measurements, training of assessors) and a description of study instruments (eg, questionnaires, laboratory tests) along with their reliability and validity, if known. Reference to where data collection forms can be found, if not in the protocol	9,10__________
	18b	Plans to promote participant retention and complete follow-up, including list of any outcome data to be collected for participants who discontinue or deviate from intervention protocols	9,10__________

Data management	19	Plans for data entry, coding, security, and storage, including any related processes to promote data quality (eg, double data entry; range checks for data values). Reference to where details of data management procedures can be found, if not in the protocol	9,10__________
Statistical methods	20a	Statistical methods for analysing primary and secondary outcomes. Reference to where other details of the statistical analysis plan can be found, if not in the protocol	9,10__________
	20b	Methods for any additional analyses (eg, subgroup and adjusted analyses)	9,10__________
	20c	Definition of analysis population relating to protocol non-adherence (eg, as randomised analysis), and any statistical methods to handle missing data (eg, multiple imputation)	______________
**Methods: Monitoring**			
Data monitoring	21a	Composition of data monitoring committee (DMC); summary of its role and reporting structure; statement of whether it is independent from the sponsor and competing interests; and reference to where further details about its charter can be found, if not in the protocol. Alternatively, an explanation of why a DMC is not needed	nil___________
	21b	Description of any interim analyses and stopping guidelines, including who will have access to these interim results and make the final decision to terminate the trial	nil__________
Harms	22	Plans for collecting, assessing, reporting, and managing solicited and spontaneously reported adverse events and other unintended effects of trial interventions or trial conduct	6_____________
Auditing	23	Frequency and procedures for auditing trial conduct, if any, and whether the process will be independent from investigators and the sponsor	nil___________
**Ethics and dissemination**			
Research ethics approval	24	Plans for seeking research ethics committee/institutional review board (REC/IRB) approval	n/a__________
Protocol amendments	25	Plans for communicating important protocol modifications (eg, changes to eligibility criteria, outcomes, analyses) to relevant parties (eg, investigators, REC/IRBs, trial participants, trial registries, journals, regulators)	n/a__________

Consent or assent	26a	Who will obtain informed consent or assent from potential trial participants or authorised surrogates, and how (see Item 32)	6_____________
	26b	Additional consent provisions for collection and use of participant data and biological specimens in ancillary studies, if applicable	6_____________
Confidentiality	27	How personal information about potential and enrolled participants will be collected, shared, and maintained in order to protect confidentiality before, during, and after the trial	9,10__________
Declaration of interests	28	Financial and other competing interests for principal investigators for the overall trial and each study site	11____________
Access to data	29	Statement of who will have access to the final trial dataset, and disclosure of contractual agreements that limit such access for investigators	9,10__________
Ancillary and post-trial care	30	Provisions, if any, for ancillary and post-trial care, and for compensation to those who suffer harm from trial participation	n/a___________
Dissemination policy	31a	Plans for investigators and sponsor to communicate trial results to participants, healthcare professionals, the public, and other relevant groups (eg, via publication, reporting in results databases, or other data sharing arrangements), including any publication restrictions	2___________
	31b	Authorship eligibility guidelines and any intended use of professional writers	nil___________
	31c	Plans, if any, for granting public access to the full protocol, participant-level dataset, and statistical code	nil___________
**Appendices**			
Informed consent materials	32	Model consent form and other related documentation given to participants and authorised surrogates	supplementary_
Biological specimens	33	Plans for collection, laboratory evaluation, and storage of biological specimens for genetic or molecular analysis in the current trial and for future use in ancillary studies, if applicable	Supplementary_

*It is strongly recommended that this checklist be read in conjunction with the SPIRIT 2013 Explanation & Elaboration for important clarification on the items. Amendments to the protocol should be tracked and dated. The SPIRIT checklist is copyrighted by the SPIRIT Group under the Creative Commons "Attribution-NonCommercial-NoDerivs 3.0 Unported" license.

For peer review only - http://bmjopen.bmj.com/site/about/guidelines.xhtml

BMJ Open

DIALOR (DIgitAL cOaching for fRailty): Protocol for a single-arm mixed methods feasibility study of a digital health coaching intervention for older people with frailty in primary care.

Journal:	*BMJ Open*
Manuscript ID	bmjopen-2023-080480.R1
Article Type:	Protocol
Date Submitted by the Author:	08-Feb-2024
Complete List of Authors:	Sait, Matthew; Bournemouth University Faculty of Health and Social Sciences, Rehabilitation and Sport Sciences<br>Christie, Rachel; Bournemouth University, Rehabilitation and Sports Sciences<br>Cox, Chantel; Bournemouth University, Nursing Science<br>Board, Michele; Bournemouth University, Nursing Science<br>Thomas, Sarah; Bournemouth University , Faculty of Health and Social Sciences<br>O'Sullivan, Cheryl; NHS Dorset<br>Davies, Cheryl; Wessex Academic Health Science Network<br>Walker, Dawn-Marie; University of Southampton, Faculty of Health Sciences<br>Vassallo, Michael; University Hospitals Dorset NHS Foundation Trust, Older Person Medicine<br>Sadler, EA; University of Southampton, Faculty of Environmental and Life Sciences, School of Health Sciences<br>Allen-Pick, Mark; Bournemouth University, Rehabilitation and Sports Sciences<br>Moore, Patrick; The Adam Practice<br>Bradbury, Katherine; University of Southampton, Psychology<br>Murphy, Jane; Bournemouth University, Humans Sciences & Public Health
**Primary Subject Heading**:	Geriatric medicine
Secondary Subject Heading:	Health informatics, General practice / Family practice, Health services research, Qualitative research
Keywords:	Aging, Health informatics < BIOTECHNOLOGY & BIOINFORMATICS, Chronic Disease, Primary Health Care, Frailty

SCHOLARONE™
Manuscripts

Title	DIALOR (DIgitAL cOaching for fRailty): Protocol for a single-arm mixed methods feasibility study of a digital health coaching intervention for older people with frailty in primary care.
**Word count**<br>(Excluding title page, abstract, references, figures, tables, or acknowledgements)	3703
**Key Words**	Aging, frailty, chronic disease, long-term conditions, health informatics, primary health care
**Authors' names**	Matthew Sait (0000-0003-0226-0351), Rachel Christie, Chantel Cox (0009-0000-7241-3179), Michele Board (0000-0002-6732-0151), Sarah Thomas (0000-0002-9501-9091), Cheryl Davies, Dawn-Marie Walker (0000-0003-2135-1363), Michael Vassallo, Euan Sadler (0000-0003-3827-224X), Cheryl O'Sullivan, Mark-Allen Pick, Patrick Moore (0000-0001-8279-6724), Katherine Bradbury (0000-0001-5513-7571), Jane Murphy (0000-0003-3531-5566)
**Author addresses, names, and positions**	Bournemouth University, 12 St Paul's Ln, BH8 8GP, Bournemouth, England Department of rehabilitation and sports science Matthew Sait Post-Graduate Researcher and Research Project Officer, Bournemouth University Rachel Christie research assistant, department of rehabilitation and sports science, Bournemouth University Chantel Cox senior lecturer in adult nursing department of nursing science, Bournemouth University Michele Board associate professor, Wessex AHSN Cheryl Davies senior programme manager innovation adoption, Bournemouth University Sarah Thomas deputy director Bournemouth university clinical research unit, University of Southampton Dawn-Marie Walker associate professor, University Hospitals Dorset Michael Vassallo consultant geriatrician, University of Southampton Euan Sadler associate professor, National Health Service (NHS) Dorset Cheryl O'sullivan advanced nurse practitioner, University Hospitals Dorset Mark-Allen Pick occupational therapist, The Adam Practice Patrick Moore GP Partner, University of Southampton Katherine Bradbury principal research fellow, Bournemouth University Jane Murphy deputy dean of research
**Corresponding author**	Correspondence to: M Sait on saitm@bournemouth.ac.uk

Abstract (300 words)

For peer review only - http://bmjopen.bmj.com/site/about/guidelines.xhtml

Background

Multi-domain interventions in older adults offer the best opportunity to prevent, delay, or reverse existing symptoms in the earlier stages of frailty and improve independence but can be costly, and difficult to deliver at scale. However, digital health interventions enable personalised care and empowerment through self-management of long-term conditions, used at any time and when combined with health coaching offer potential to enhance wellbeing and facilitate achievement of health-related goals. We aim to evaluate the feasibility and acceptability of a digital health platform for long-term disease management combined with health coaching for people living with mild-moderate frailty, targeting self-identified goals – activity, nutrition, mood, enhancing social engagement and well-being.

Methods and analysis

This is a non-randomised feasibility, single group, pre-test/post-test study, using qualitative and quantitative methods. The digital health coaching intervention (DIALOR - DIgitAL cOaching for fRailty) has been developed for implementation to older adults, aged 65 years or older with mild to moderate frailty and diagnosis of one or more long-term health conditions in the community. Participants will receive 12 weeks of health coaching and have access to a mobile health platform for 6 months. The primary outcome measure is the acceptability and feasibility of DIALOR along with a range of secondary outcome measures (including frailty, functioning measures, quality of life, social engagement, diet quality and self-reported indicators) collected at baseline and at 6 months. The findings will inform whether a wider effectiveness trial is feasible and if so, how it should be designed.

Ethics and Dissemination

Ethical approval has been granted by the Southeast Scotland Research Ethics Committee 02 (reference: 22/SS/0064). Research findings will be disseminated in a range of different ways to engage different audiences, including publishing in open-access peer-reviewed journals, conference presentations, social media, dissemination workshop with patients, carers, and healthcare professionals and on institution websites.

For peer review only

Article Summary

Strengths and limitations of this study

- A strength of the study design is using a mixed methods approach with qualitative understanding of the implementation process and quantitative measures to assess the effects on health outcomes and whether the trial is feasible.
- There is strong involvement of stakeholders and people with the lived experience to develop and guide the study.
- Potential limitations are as a feasibility study, it will assess a pre- and post-test intervention with non-randomisation of sample and to sufficiently engage hard-to reach target groups including those with poor digital literacy and low socio-economic status, impacting representation of the participant group and generalisability of the findings.

Introduction

For peer review only - http://bmjopen.bmj.com/site/about/guidelines.xhtml

Frailty is a multidimensional syndrome characterised by cumulative loss of reserves and accumulation of deficits (e.g., energy, physical ability, cognition, social and psychological aspects) that give rise to vulnerability and risk of adverse outcomes with advancing age (1). It is not an inevitable consequence of ageing but older adults with frailty have increased risk of poor health, including falls, fractures and functional impairment, hospitalisation, need for nursing home care, and increased mortality (2). Older people with frailty experience impairment in their activities of daily living and often report significant reduction in their quality of life (3). Furthermore, studies have shown that frailty may be related to decreased mood (4) and may be shaped by social factors such as different levels of social support or living alone (5). Frailty affects about 12% of people aged ≥ 65 years worldwide (6) and 14% of older people in the United Kingdom (UK) (7), and even higher numbers of individuals in the early stages of frailty, ranging from 19-53% (8). Frailty is an ever-present burden amongst international populations, which has been exacerbated by the impact of the Covid-19 pandemic (9) and the resultant deconditioning (10).

Whilst moderate to severe frailty has a higher risk of physical health declines, mild-moderate frailty with some loss of physiological reserve is considered to be potentially reversible (11) to a robust or stable state (12). Mild frailty is conceptualised as an intermediate stage on the Rockwood Clinical Frailty Scale (13), whereby older people experience some loss of physiological reserves but can usually recover after a negative stressor event, such as an infection or fall. This would indicate that interventions targeting earlier stages of frailty are more likely to reverse frailty decline. Moreover, there is evidence to suggest that interventions, particularly multi-domain interventions in older adults including mobility, strength, balance, nutrition, physical activity, and social support offer the best opportunity to prevent, delay, or reverse existing symptoms of frailty and improve independence (14, 15, 16, 17). Most multi-component interventions incorporate involvement from a multidisciplinary team of health and social care professionals meaning they can be costly, and difficult to deliver at scale (18).

Digital health interventions (DHIs) enable personalised care and empowerment through self-management of a long-term condition, which can be used at any time, and may offer more cost-effective health care delivery (19). DHIs have been shown to be effective at achieving successful remote monitoring and management of COPD amongst older populations (20), suggesting that DHIs may be a feasible method of reaching remote communities. Studies show that digital approaches for lifestyle modifications in other long-term conditions such as Type 2 diabetes, offer effective and scalable options when face-to face or in-person programmes are not accessible or feasible, especially when offered with health coaching (21, 22).

Health Coaching was originally described as the practice of health education and health promotion within a coaching context, to enhance the wellbeing of individuals and to facilitate achievement of health-related goals (23). More recently, HC has been defined as helping people gain and use the knowledge, skills, and confidence to become active participants in their care so that they can reach their self-identified health and wellbeing goals (24). In the last decade, HC has become increasingly embedded within Integrated Care Services. However, the evidence base demonstrating the effectiveness of HC in the UK is limited. For example, a systematic review and meta-analysis assessing the impact of HC on Type 2 diabetes found that HC was effective in achieving optimal glycaemic levels via self-management strategies (25). In the UK, health coaches working within

For peer review only - http://bmjopen.bmj.com/site/about/guidelines.xhtml

primary care can be an established clinical team member (e.g., registered nurses, physician associates, physiotherapists, paramedics, occupational therapists, and psychologists) or can be unlicensed health workers (26). Furthermore, research is emerging to show that HC may offer a promising approach to support effective lifestyle interventions for managing chronic conditions such as asthma, diabetes, cardiovascular disease, and mental illness on an international stage (27).

Current Integrated Care Systems in the UK have also implemented both DHIs via mHealth and HC to improve self-management of their long-term conditions (LTC), amongst older people living with COPD, diabetes, and cardiovascular disease (28). However, there remains a distinct lack of evidence which demonstrates the effectiveness of HC for older adults who have at least two chronic conditions (multimorbidity), which is thought to affect up to 50% of older people (aged over 65 years) (29, 30). Further, the impact of HC for older adults with a diagnosis of frailty, is unknown, making a single disease focused model of healthcare unsuitable to provide effective management (31). Therefore, targeted behaviour change interventions using DHIs such as mHealth, in conjunction with HC, have potential to offer a proactive, and wide-reaching approach to prevent frailty decline alongside the management of other diagnosed co-morbidities and improve health and wellbeing outcomes.

This study aims to address this gap by co-designing and implementing a multicomponent intervention for people living with mild-moderate frailty. The DIALOR intervention will include two key components (Figure 1);

1) Digital health coaching
    - This will include 12 weeks of digital HC with 6 sessions in total. Each session will be conducted fortnightly and will have a duration of 45 minutes – 1 hour. Each session will be conducted by a qualified health coach. An online platform (Zoom/Accurx/Microsoft Teams) will be used to deliver the digital HC intervention.
2) Mobile health platform
    - A suite of web-based platforms offering tailored clinical advice and support and self-management resources for management of chronic obstructive pulmonary disease (COPD), Asthma, cardiovascular disease (CVD) and type 2 diabetes will be used throughout the study (details of the online platform can be accessed via https://mymhealth.com/). Each participant will be provided access to the mobile health platform which relates to their diagnosed LTC. The platforms can be accessed by any electronic device (e.g., smartphone, tablet, or television) that has an active internet connection.

The primary objectives of this study are to:

- Explore barriers and facilitators of the digital intervention from the perspectives of health coaches and healthcare professionals (HCPs) and determine the essential and desirable features of the intervention (wants and needs; preferred outcomes) to identify what functions will be most useful for the target population.
- Explore how HC can provide support for the management of mild-moderate frailty and long-term conditions from the perspectives of older people with mild-moderate frailty, carers, HCPs, and health coaches.

For peer review only - http://bmjopen.bmj.com/site/about/guidelines.xhtml

- Determine the acceptability and feasibility of the intervention for older adults with mild/moderate frailty and another long-term condition referred to access health coaching services and willing to engage with the mobile health platform.

The secondary objectives of this study are:

- Explore the factors which inhibit or facilitate implementation including contextual factors, recruitment processes, resources needed, potential mechanisms and pathways to impact.
- Explore the acceptability and suitability of outcomes measures (to inform sample size estimation and determine the primary outcome measure) for a future randomised controlled trial (RCT) of the intervention (should it be feasible).
- Measure key domains for secondary outcome measures including participants who are able to self-report measures.

Figure 1: Overview of the DIALOR intervention and its functionalities

Methods and Analysis

Intervention Development

Over a 9-month period, intervention development will use guiding principles (32) to inform co-design objectives of a logic model (Figure 2) and ensure the intervention is acceptable, feasible, engaging, and easy to implement. A systematic review and narrative synthesis will also guide the development of key features our intervention requires to meet user needs and best practice guidelines.

Behavioural analysis (33) will triangulate evidence from the i. the narrative synthesis, ii. consultation with two Stakeholder Engagement Groups (SEG) including HCPs, health coaches, commissioners of service and patient and iii. public involvement (PPI) advisors, as well as iv. a separate PPI group with lay advisors to identify barriers in each domain - activity/mobility, nutrition, social engagement, and psychological well-being. Intervention components addressing each barrier will be theoretically mapped and characterised using the Behaviour Change Wheel (BCW) (34), the Theoretical Domains Framework (TDF) (35) and Normalisation Process Theory (NPT). NPT is the process through which complex, new ways of working become embedded into standard practice (36). We will use the Behaviour Change Techniques Taxonomy Version 1 (BCTT v1) (37) to code the behaviour change techniques delivering the identified intervention functions. This will provide a clear description of the intervention and factors which may influence its implementation and will be summarised in a behavioural analysis table. We will then convene another SEG meeting to discuss and refine the proposed logic model.

To identify challenges/enabling factors for delivery/implementation and intervention modifications needed to maximise engagement and behaviour change, we will conduct individual interviews with people with mild to moderate frailty (and their carers where appropriate) (n=20) and focus groups (n=4) with HCPs and health coaches (n=20). These will be conducted face-to-face, and audio recorded using dictaphones, otherwise they will be conducted and recorded through Microsoft Teams or Zoom. Final in-house testing of the intervention will be undertaken before the commencement of the study. We will use the TIDieR checklist (38) for intervention description which will report relevant outcomes to inform further planning for a future trial.

Study Design

For peer review only - http://bmjopen.bmj.com/site/about/guidelines.xhtml

This feasibility study is a non-randomised, single group, pre-test/post-test study, using qualitative and quantitative methods. This protocol was developed in accordance with the Standard Protocol Items: Recommendations for Intervention Trials (SPIRIT) reporting guidelines (39).

This will be a mixed methods prospective cohort study conducted within primary care settings in Southwest England. Firstly, online group training of the intervention will be provided for health coaches by the research team, with top-up training and support as necessary throughout. A training manual will also incorporate frailty awareness, and the CARE (Congratulate, Ask, Reassure, Encourage) model approach (40, 41). Intervention delivery will be tailored to each patient, with goals and strategies to help achieve them developed in conjunction with the health coach.

Study setting
The study will work with GP practices in Primary Care Networks (PCN) in Southwest England to identify patients already using the mobile health platform. GP practices will be eligible to take part if they are currently referring older people with mild to moderate frailty to access HC/social prescribing services and willing to test new digital approaches.

Participant recruitment
Participant recruitment will be conducted within primary care services, at individual participating GP surgeries. Up to 50 older people with mild-moderate frailty will be recruited in line with previous sample size recommendations for feasibility studies (42). Eligibility criteria for older adults with mild to moderate frailty to participate in the study are outlined in Table 1. The clinical care team from participating GP surgeries will identify eligible participants through existing medical records.

Three strategies will be utilised to recruit patients i) via text message invitation to eligible patients which provides a link to a website with further information about the study, participant information sheet and contact information for the research team. Potential participants will be followed up by the research team to provide an opportunity to answer questions, confirm eligibility and obtain informed consent to participate, ii) by a member of the research team approaching eligible participants at the GP surgery, signposted by the clinical team and iii) via leaflets provided to eligible patients by the clinical team.

Table 1: Inclusion and exclusion criteria

Inclusion criteria
• Aged ≥ 65 years<br>• Scoring as mild or moderately frail using the Electronic Frailty Index<br>• Diagnosis of 1 or more long-term conditions including COPD, Type 2 diabetes, heart disease or asthma<br>• Living in their own homes<br>• Capacity to consent to participate<br>• Able to communicate fluently enough in English to take part in the study<br>• Able to recall their experiences sufficiently to engage with a health coach<br>• Self-reported ability to use digital technology independently or with support
Exclusion criteria
• Nursing home resident or on active waiting list for a place<br>• Individuals who have a terminal illness and are being managed for end-of-life needs<br>• Lacking capacity to consent (e.g., advanced dementia)

Figure 2: Proposed logic model

For peer review only - http://bmjopen.bmj.com/site/about/guidelines.xhtml

Figure 3: Process flow of study design

Digital Support for patients

Patients with low digital literacy will be supported by the research team to access the digital HC and mobile health platforms. They will be offered a training manual, developed by the research team, and co-produced alongside the SEG and PPI group alongside digital support leaflets and guidance on keeping safe online. To reduce the risk of digital inequity, internet enabled tablets may be provided to individuals who lack a device, for the length of the study. Internet and data packages (via local council schemes) will be provided if the participant lacks access to an established internet connection. Digital support will be offered throughout the study via local council services (volunteer digital upskilling clinics embedded within public spaces e.g., GP surgeries and council libraries) and the research team.

Outcome measures

Primary outcomes

The primary outcome measures are feasibility and acceptability of the DIALOR intervention. Feasibility will be determined from the number of health coaches trained, number of participants recruited, retention and adherence to the intervention as well as any adverse events. To determine the acceptability of the intervention and to explore barriers and enablers to the implementation of the intervention, semi-structured interviews will be conducted with patients with mild-moderate frailty (and their carers where appropriate) (n= 20), HCPs and health coaches (n= 20) who participated in the study.

The interviews will consist of open-ended questions to explore participant views of the intervention, barriers, and facilitators to inform the design of further studies. To understand the factors influencing implementation, the interview schedule will be underpinned by normalisation process theory (NPT) (43). NPT is an implementation theory providing a framework to identify and explain important elements of the implementation process. As such, it informs an understanding of the social processes through which new or practice can be implemented, embedded, and integrated into care settings.

Secondary outcomes

The secondary outcomes will include the measurement of functioning, quality of life, physical activity levels, frailty severity, diet quality, and engagement using validated outcome measures. Physical functioning, a key outcome in frailty trials (44), will be measured using the Modified Barthel Index (MBI) (45) and Lawton-Brody Instrumental Activities of Daily Living (IADL) (46). The MBI will measure older adults' functional ability using 10 items, with a higher score reflecting higher ability to function independently. The MBI has been found to be of reasonable reliability when used amongst older adults (47). The IADL will measure more complex activities of daily living using 8 key domains. The International Physical Activity Questionnaire short form (IPAQ-SF) will measure older adults activity levels in the last 7 days (48). The evidence supporting the IPAQ-SF as an indicator of relative or absolute physical activity is weak (49). However, it is a validated generalised outcome measure for older adult populations (65 years or over) (50).

For peer review only - http://bmjopen.bmj.com/site/about/guidelines.xhtml

Quality of life is a core outcome in frailty trials (51) and will be measured using the EuroQol-5 dimensions-5 levels (EQ-5D-5L) questionnaire which includes 5 health areas (mobility, self-care, usual activities, pain/discomfort, and anxiety/depression). These 5 health areas are assessed with 5 main response levels (no problems, slight, moderate, severe, or unable), along with a visual analogue scale (52).

Frailty severity will be measured using the Groningen Frailty Indicator (GFI) (53). The GFI is a multidimensional screening instrument consisting of 15 self-report items. It is a feasible and widely used screening instrument for identifying frail older adults (54, 55). Higher GFI scores indicate higher frailty levels and an increased need for integrated care (56).

The Short Form Food Frequency Questionnaire (SFFFQ) will measure dietary quality. The SFFQ asks about foods which respondents might have consumed, on average, over a set time period (3 months) and is found to be an effective instrument for assessing diet quality in large scale studies with UK adult populations (57).

Social engagement will be measured using the Lubben Social Network Scale (LSNS-6) and mental wellbeing will be measured using the Warwick-Edinburgh Mental Well-Being Scale (WEMWBS) Self-reported indicators such as height, weight, BMI (Body Mass Index), number of falls (per year), goal attainment, alcohol intake and smoking habits (if applicable) will be measured at baseline (pre-intervention), throughout the study during digital HC intervention, and at 6 months (post-intervention).

Data will be collected from the mobile health platform which will be recorded automatically by inbuilt tracking software, including usage patterns from number of logins, and length of time that older people and their carers use the platform. These data will be collected at 6 months for each patient participant (post-intervention).

Data Analysis

Quantitative data analysis

Data will be entered into a secure database for analyses. Statistical analyses will be conducted using statistical software SPSS (V.28.0). Descriptive statistics: median (IQR); mean (SD); number (%) and confidence interval – will be used to analyse the number of volunteers recruited and retained, as well as participants' adherence to the intervention and pre-post intervention outcome measures to assess the feasibility of delivering the intervention. Pre- and post-analyses at 6 months, will be conducted to determine if the intervention had an impact on the lifestyle management of frailty measured by functioning (MBI, IADL), social engagement (LSNS-6), mental wellbeing (WEMWBS) quality of life (EQ-5D-5L), physical activity (IPAQ-SF), frailty indicator (GFI), diet quality (SFFFQ) and self-reported indicators.

Qualitative data analysis

Data collected from all the interviews and focus groups will be transcribed verbatim and analysed using thematic analysis (TA). TA is a commonly used method for identifying, analysing, and reporting patterns or themes within data (58). There are six key phases: Phase 1 – familiarisation with the data, Phase 2 – initial code generation, Phase 3 – searching for and generating themes, Phase 4 –

For peer review only - http://bmjopen.bmj.com/site/about/guidelines.xhtml

reviewing themes, Phase 5 – defining and naming themes and Phase 6 – producing the report. Analysis of qualitative data will be conducted using Microsoft word, and or alternative software such as NVivo (V.12). RC will analyse codes using NPT (43) and the Behaviour Change Technique Taxonomy (37) to focus on the social processes shaping the implementation process from making sense of the intervention to engaging in it (individually and collectively) and embedding this (normalising) in everyday practice (or people's everyday lives) and to identify the facilitators and barriers present throughout the implementation process. Themes will be developed, from the codes, to reflect the lived experience and views of patient participants, health coaches, and HCPs regarding the digital HC and mobile health platform intervention. MS will code 25% of interviews and focus groups separately to develop, discuss, and agree themes with RC. Finally, members of the core research team (ES, MB, CC, JM) will be consulted to develop, discuss, and agree themes via an iterative process.

Patient and Public Involvement

Public/patient involvement and engagement (PPI) has been and will continue to be part of all stages of the research from developing the proposal to providing feedback on preliminary findings, to dissemination and impact, and input for tasks such as finalising interview/focus group topic guides. For the development of this programme of research, we consulted with seven older adults and family carers who helped inform the proposal. They thought that this was an important topic and liked the HC approach to support digital engagement. We have convened a PPI advisory group (6-10 participants, aged 60+ years, meeting 3 to 4 times throughout the project). A PPI representative as a co-researcher leads the PPI group and attends the project advisory group. The group represents the views of people from different socio-economic backgrounds including those who do and do not have access to various online and digital technologies.

PPI meetings will be face-to-face, and advisors will be offered reimbursement aligning with National Institution for Health and Care Research (NIHR) payment guidance. Advisors will have the opportunity to contribute via email and phone between meetings. Advisors will be sent materials prior to meetings and able to discuss matters arising from meetings afterwards. Advisors will be provided with meeting summaries and feedback on how their involvement informed the research project. A PPI plan will be developed and shaped by PPI and results/activities will be shared with the project advisory group. Monitoring and reporting will use the GRIPP checklist (59).

Ethics and dissemination

The DIALOR project is sponsored by Bournemouth University. Ethical approval has been granted by the Southeast Scotland Research Ethics Committee 02 (reference: 22/SS/0064). Data collected during the study will be pseudonymised by a member of the research team (RC) to ensure removal of identifying information from research data prior to data analysis. Data will be stored on Bournemouth University servers, and identification data will be stored in encrypted files in a separate, secure location. Data transfer will be undertaken through secure, encrypted emails or Bournemouth University's secure file transfer system (BU Transfer). We will publish results in peer-reviewed open access journals. A lay summary of the final study report will be accessible on the project website:
(https://www.bournemouth.ac.uk/research/projects/dialor-digital-coaching-frailty). We will also seek to present our findings at conferences, workshops for patients, write lay articles for

For peer review only - http://bmjopen.bmj.com/site/about/guidelines.xhtml

dissemination to the public, and share the findings through our networks across health and social care.

Status

The trial commenced September 2023 and is expected to be completed by September 2024.

Acknowledgements

The authors thank study participants and patient advisers for their input. The authors wish to thank GP director Simone Yule for helping to implement the study within local primary care services. The authors would like to thank colleagues from Dorset Help & Care charity and my mhealth for helping to implement the study intervention and imbed this seamlessly within primary care services.

Author Contributions

The study concept and design was conceived by JM. MS, RC, ES, CC, and MB assisted in refining the study questionnaires and study design. MS, RC, and MAP will be responsible for data collection. Analyses will be conducted by MS, RC, ES, DMW, CS, KB, CC, MB, JM, and MAP. MS prepared the first draft of the manuscript. All authors critically revised the manuscript and approved the submitted version. The corresponding author attests that all listed authors meet authorship criteria and that no others meeting the criteria have been omitted.

Funding

This research was support by NIHR Applied Research Collaborative Wessex, grant number (ARC 001).

Disclaimer

Provenance and peer review not commissioned; externally peer reviewed.

Competing interests

None declared.

Copyright/license

The Corresponding Author has the right to grant on behalf of all authors and does grant on behalf of all authors, a worldwide licence to the Publishers and its licensees in perpetuity, in all forms, formats and media (whether known now or created in the future), to i) publish, reproduce, distribute, display and store the Contribution, ii) translate the Contribution into other languages, create adaptations, reprints, include within collections and create summaries, extracts and/or, abstracts of the Contribution, iii) create any other derivative work(s) based on the Contribution, iv) to exploit all subsidiary rights in the Contribution, v) the inclusion of electronic links from the Contribution to third party material where-ever it may be located; and, vi) licence any third party to do any or all of the above.

For peer review only - http://bmjopen.bmj.com/site/about/guidelines.xhtml

For peer review only

References

1. Rockwood, K., Song, X., MacKnight, c., Bergman, H., Hogan, D., McDowell, I., Mitnitski, A., 2005. A global clinical measure of fitness and frailty in elderly people, *Canadian Medical Association Journal*, 173 (5), 489-495.
2. Clegg, A., Young, J., Iliffe, S., Rikkert, M., Rockwood, K., 2013. Frailty in elderly people, *Lancet*, 381 (9868), 1-25.
3. Langlois, F., Vu, T., Chasse, K., Dupuis, G., Kergoat, M J., Bherer, L., 2012. Benefits of physical exercise training on cognition and quality of life in frail older adults, The *Journals of Gerontology, Series B, Psychological Sciences and Social Sciences,* 68 (3), 400-404.
4. Collard, R., Comijs, H., Naarding, P., Penninx, B., Milaneschi, Y., Ferruci, L., Voshaar, R., 2015. Frailty as a Predictor of the Incidence and Course of Depressed Mood, *Journal of the American Medical Directors Association*, 16 (6), 509-514.
5. Robertson, D., Savva, G., Kenny, R., 2013. Frailty and cognitive impairment – A review of the causal mechanisms, *Ageing Research Reviews*, 12 (4), 840-851.
6. O'Caoimh, R., Sezgin, D., O'Donovan, M,R., Molloy, D,W., Clegg, A., Rockwood, K., 2021. Prevalence of frailty in 62 countries across the world: a systematic review and meta-analysis of population-level studies, Age Ageing, 50 (1), 96–104.
7. Gale, C., Cooper, C., Deary I., Sayer A., 2014. Psychological well-being and incident frailty in men and women: the English Longitudinal Study of Ageing, *Psychological Medicine*, 44 (4), 697-706.
8. Collard, R., Boter, H., Schoevers, R., Voshaar, R., 2012. Prevalence of Frailty in Community-Dwelling Older Persons: A Systematic Review, *Journal of the American Geriatrics Society*, 60 (8), 1487-1492.
9. Hussien, H., Nastasa, A., Apetrii, M., Nistor, I., Petrovic, M., Covic, A., 2021. Different aspects of frailty and COVID-19: points to consider in the current pandemic and future ones, *BMC Geriatrics*, 21 (389), 1-11.
10. Steinman, M., Perry, L., Perissinotto, C., 2020. Meeting the Care Needs of Older Adults Isolate at Home During the COVID-19 Pandemic, *JMA Internal Medicine*, 180 (6), 819-820.
11. Ng, T., Feng, L., Nyunt, M., Feng, L., Niti, M., Tan, B., Chan, G., Khoo, S., Chan, S., Yap, P., Yap, K., 2015. Nutritional, Physical, Cognitive, and Combination Interventions and Frailty Reversal Among Older Adults: A Randomized Controlled Trial, *The American Journal of Medicine*, 128 (11), 1225-1236.
12. Gill, T., Gahbauer, E., Allore, H., Han L., 2006. Transitions between frailty states among community-living older persons, *Archives of Internal Medicine*, 166 (4), 418-423.
13. Rockwood, K., Theou, O., Mitnitski, A., 2015. What are frailty instruments for? *Age and Ageing*, 44, 545-547.
14. Cameron, I., Fairhall, N., John, B., Lockwood, K., Monaghan, N., Sherrington, C., 2017. A multifactorial interdisciplinary intervention in pre-frail older people: randomised trial, *Innov Aging*, 1 (196)
15. Walters, K., Frost, R., Kharicha, K., Avgerinou, C., Gardner, B., Ricciardi, F., 2017. Home-based health promotion for older people with mild frailty: the Home Health intervention development and feasibility RCT, *Health Technol Assess*, 21 (73), 1–128.

For peer review only - http://bmjopen.bmj.com/site/about/guidelines.xhtml

16. Serra-Prat, M., Sist, X., Domenich, R., Jurado, L., Saiz, A., Roces, A., 2017. Effectiveness of an intervention to prevent frailty in pre-frail community-dwelling older people consulting in primary care: a randomised controlled trial, Age Ageing, 46 (3), 401–7.
17. Yu, R., Tong, C., Ho, F., Woo, J., 2020. Effects of a Multicomponent Frailty Prevention Program in Prefrail Community-Dwelling Older Persons: A Randomized Controlled Trial. J Am Med Dir Assoc, 21 (2), e1- e10.
18. Chen, Z., Ding, Z., Chen, C., Sun, Y., Jiang, Y., Liu, F., Wang, S., 2021. Effectiveness of comprehensive geriatric assessment intervention on quality of life, caregiver burden and length of hospital stay: a systematic review and meta-analysis of randomised controlled trials, *BMC Geriatrics*, 21(1), 377.
19. Gomes, M., Murray, E., Raftery, J., 2022. Economic Evaluation of Digital Health Interventions: Methodological Issues and Recommendations for Practice, *PharmacoEconomics*, 40, 367-378.
20. Velardo, C., Shah, S., Gibson, O., Clifford, G., Heneghan, C., Rutter, H., Farmer, A., Tarassenko, L., 2017. Digital health system for personalised COPD long-term management, *BMC Medical Informatics and Decision Making*, 17 (19), 1-13.
21. Smith, A., Crippa, A., Woodcock, J., Brage, S., 2016. Physical activity and incident type 2 diabetes mellitus: a systematic review and dose-response meta-analysis of prospective cohort studies, *diabetologia*, 59 (12), 2527-2545.
22. Castro Sweet, C., Chiguluri, V., Gumpina, R., Abbott, P., Madero, E., Payne, M., Happe, L., Matanich, R., Renda, A., Prewitt, T., 2018. Outcomes of a Digital Health Program With Human Coaching for Diabetes Risk Reduction in a Medicare Population, *Journal of Aging and Health*, 30 (5), 692-710.
23. Palmer, S., Cooper, C., Thomas, K., 2003. Creating a Balance: Managing Stress. London: British Library.
24. NHS England 2019. Universal Personalised Care: Implementing the Comprehensive Model.
25. Pirbaglou, M., Katz, J., Motamed, M., Pludwinski, S., Walker, K., Ritvo, P., 2018. Personal Health Coaching as a Type 2 Diabetes Mellitus Self-Management Strategy: A Systematic Review and Meta-Analysis of Randomized Controlled Trials, *American Journal of Health Promotion*, 32 (7), 1613-1626.
26. Gastala, N., Wingrove, P., Gaglioti, A., Liaw, W., Bazemore, A., 2018. The Growing Trend of Health Coaches in Team Based Primary Care Training, *Family medicine*, 50 (7), 526-530.
27. Yang, J., Bauer, B., Lindeen, S., Perlman, A., Dabrh, A., Boehmer, K., Salinas, M., Cutshall, S., 2020. Current trends in health coaching for chronic conditions., *Open Medicine*, 99 (30), 1-6.
28. McShane, M. and Kirkham, K. (2020). Making it personal – population health management and the NHS", *Journal of Integrated Care*, 28 (3), 243-252.
29. Barnett, K., Mercer, SW., Norbury, M., Watt, G., Wyke, S., Guthrie, B., 2012. Epidemiology of multimorbidity and implications for health care, research, and medical education: a cross-sectional study. *Lancet 2012*; 380 (9836), 37–43.
30. Salive, ME., 2013. Multimorbidity in older adults. *Epidemiol Rev*, 35, 75–83.
31. Kingston, A., Robinson, L., Booth, H., Knapp, M., Jagger, C., 2018. Projections of multi-morbidity in the older population in England to 2035: estimates from the Population Ageing and Care Simulation (PACSim) model, *Age and Ageing*, 47 (3), 374-380.
32. Yardley, L., Morrison, L., Bradbury, K., Muller, I., 2015. The person-based approach to intervention development: application to digital health-related behavior change interventions, *Journal of medical Internet research*, 17 (1), 1-15.

For peer review only - http://bmjopen.bmj.com/site/about/guidelines.xhtml

33. Band, R., Bradbury, K., Morton, K., May, C., Michie, S., Mair, F., Murray, E., McManus R., Little, P., Yardley, L., 2017. Intervention planning for a digital intervention for self-management of hypertension: a theory-, evidence- and person-based approach, *Implementation Science*, 12 (25), 1-13.
34. Michie, S., van Stralen, M.M., West, R., 2011. The behaviour change wheel: A new method for characterising and designing behaviour change interventions. *Implementation Science,* 42 (6), 1-11.
35. Cane, J., O'Connor, D., Michie, S., 2012. Validation of the theoretical domains framework for use in behaviour change and implementation research. *Implementation Science*, 37 (7), 1-17.
36. May, C., Cummings, A., Girling, M., Bracher, M., Mair, F., May, C., Murray, E., Myall, M., Rapley, T., Finch, T., 2018. Using Normalization Process Theory in feasibility studies and process evaluations of complex healthcare interventions: a systematic review, *Implementation Science*, 13 (80), 1-27.
37. Michie, S., Richardson, M., Johnston, M., Abraham, C., Francis, J., Hardeman, W., Eccles, M., Cane, J., Wood, C., 2013. The behaviour change taxonomy (v1) of 93 hierarchically clustered techniques: building an international consensus for the reporting of behaviour change interventions, *Annals of Behavioural Medicine*, 46 (1), 81-95.
38. Hoffman, T., Glasziou, P., Milne, R., Moher, D., Barbour, V., Johnston, M., Lamb, S., Dixon-Woods, M., Wyatt, J., 2014. Better reporting of interventions: a template for intervention description and replication (TIDieR) checklist and guide, *BMJ*, 348, 1-12.
39. Chan, A, W., Tetzlaff, J., Gøtzsche, P., Altman, D., Mann, H., Berlin, J., Dickersin, K., Hróbjartsson, A., Schulz, K., Parulekar, W., Krleža-Jerić, K., Laupacis, A., Moher, D., 2013. SPIRIT 2013 statement: defining standard protocol items for clinical trials, *Annals of Internal Medicine*, 158 (3), 200-207.
40. Bradbury, K., Morton, K., Band, R., May, C., McManus, R., Little, P., Yardley, L., 2017. Understanding how primary care practitioners perceive an online intervention for the management of hypertension, *BMC Medical Informatics and Decision* Making, 17 (5), 1-13.
41. Smith, E., Bradbury, K., Scott, L., Steele, M., Little, P., Yardley, L., 2017. Providing online weight management in primary care: a mixed methods process evaluation of healthcare practitioners' experiences of using and supporting patients using POWeR+, *Implementation Science*, 12 (69), 1-13.
42. Billingham, S., Whitehead, A., Julious, S., 2013. An audit of sample sizes for pilot and feasibility trials being undertaken in the United Kingdom registered in the United Kingdom Clinical Research Network database, *BMC Medical Research and Methodology*, 13 (104), 1-6.
43. May, C., Mair, F., Finch, T., MacFarlane, A., Dowrick, C., Treweek, S., Rapley, T., Ballini, L., Ong, B., Rogers, A., Murray, E., Elwyn, G., Légaré, f., Gunn, J., Montori, V., 2009. Development of a theory of implementation and integration: Normalization Process Theory, *Implementation Science*, 4 (29), 1-9.
44. Tappenden, P., Campbell, F., Rawdin, A., Wong, R., Kalita, N., 2012. The clinical effectiveness and cost-effectiveness of home-based, nurse-led health promotion for older people: a systematic review, *Health Technology Assessment*, 16 (20), 1-72.
45. Wade, DT., and Collin, C., 1988. The Barthel ADL Index: a standard measure of physical disability, *International Disability Study*, 10, 64-67.
46. Lawton, M.P., and Brody, E.M, 1969. Assessment of older people: Self-maintaining and instrumental activities of daily living. The Gerontologist, 9 (3), 179-186.

For peer review only - http://bmjopen.bmj.com/site/about/guidelines.xhtml

47. Hopman-Rock, M., Hirtum, H., Vreede, P., Freiberger, E., 2019. Activities of daily living in older community-dwelling persons: a systematic review of psychometric properties of instruments, *Aging Clinical and Experimental Research*, 31, 917-925.
48. Hagstromer, M., Oja, P., Sjostrom, M., 2006. The international physical activity questionnaire (IPAQ): a study of concurrent and construct validity. *Public Health Nutrition*, 9 (6), 755-762.
49. Lee, P., Macfarlane, D., Lam, T., Stewart, S., 2011. Validity of the international physical activity questionnaire short form (IPAQ-SF): A systematic review. *International Journal of Behavioural Nutrition and Physical Activity*, 8 (115), 1-11.
50. Hurtig-Wennlöf, A., Hagströmer, M., Olsson, LA., 2010. The International Physical Activity Questionnaire modified for the elderly: aspects of validity and feasibility. *Public Health Nutr*, 13, 1847–54.
51. Prorok, J., Williamson, P., Shea, B., Rolfson, D., Manas, L., Cesari, M., Kim, P., Muscedere, J., 2022. An international Delphi consensus process to determine a common data element and core outcome set for frailty: FOCUS (The Frailty Outcomes Consensus Project), *BMC Geriatrics*, 22 (284), 1-9.
52. The EuroQoL Group. EuroQol--a new facility for the measurement of health-related quality of life. Health Policy 1990;16:199-208.
53. Steverink, N., 2001. Measuring frailty: developing and testing the GFI (Groningen frailty Indicator). *The Gerontologist*, 41, 236.
54. Metzelthin S, Daniëls R, Van Rossum E, De Witte L, van den Heuvel W, Kempen G., 2010. The psychometric properties of three self-report screening instruments for identifying frail older people in the community. *BMC Publ Health*, 10 (1), 176–183.
55. Peters LL, Boter H, Buskens E, Slaets JPJ., 2012. Measurement properties of the groningen frailty indicator in home-dwelling and institutionalized elderly people, *J Am Med Dir Assoc*, 13, 546–551.
56. Slaets, J,P., 2006. Vulnerability in the elderly: frailty. *Med Clin North Am*, 90 (4), 593–601.
57. Cleghorn, C., Harrison, R., Ransley, J., Wilikinson, S., Thomas, J., Cade, J., 2016. Can a dietary quality score derived from a short-for FFQ assess dietary quality in UK adult population surveys? *Public Health Nutrition*, 19 (16), 2915-2923.
58. Braun, V., Clarke, V., 2014. What can " thematic analysis " offer health and wellbeing researchers? *Int J Qual Stud Health Well-Being*, 9 (26152), 1-2.
59. Staniszewska, S., Brett, J., Simera, I., Seers, K., Mockford, C., Goodlad, S., Altman, D., Moher, D., Barber, R., Denegri, S., Entwistle, A., Littlejohns, P., Morris, C., Suleman, R., Thomas, V., Tysall, C., 2017. GRIPP2 reporting checklists: tools to improve reporting of patient and public involvement in research, *BMJ*, 2017, 358, 1-7.

For peer review only - http://bmjopen.bmj.com/site/about/guidelines.xhtml

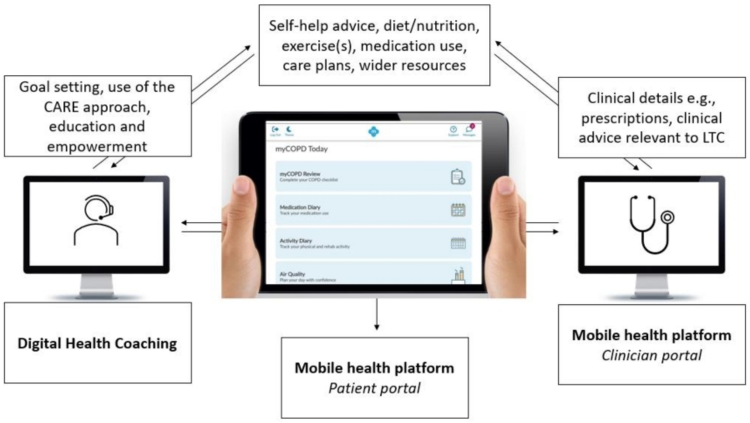

297x210mm (300 x 300 DPI)

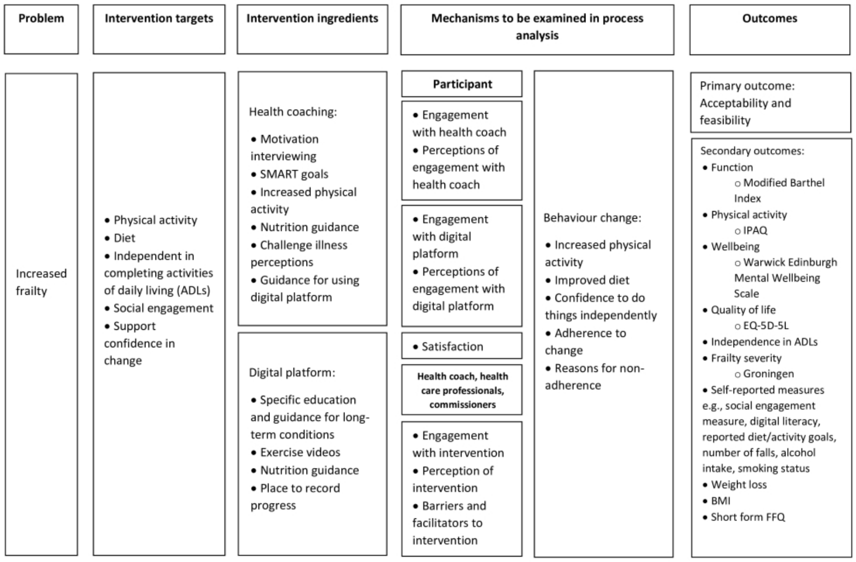

Figure 2: Proposed logic model

297x210mm (300 x 300 DPI)

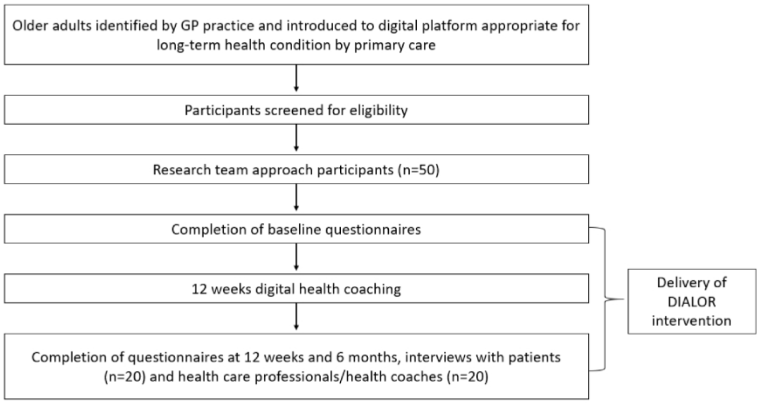

297x210mm (300 x 300 DPI)

STANDARD PROTOCOL ITEMS: RECOMMENDATIONS FOR INTERVENTIONAL TRIALS

SPIRIT 2013 Checklist: Recommended items to address in a clinical trial protocol and related documents*

Section/item	Item No	Description	Addressed on page number
**Administrative information**			
Title	1	Descriptive title identifying the study design, population, interventions, and, if applicable, trial acronym	1
Trial registration	2a	Trial identifier and registry name. If not yet registered, name of intended registry	1
	2b	All items from the World Health Organization Trial Registration Data Set	nil
Protocol version	3	Date and version identifier	1
Funding	4	Sources and types of financial, material, and other support	11
Roles and responsibilities	5a	Names, affiliations, and roles of protocol contributors	11
	5b	Name and contact information for the trial sponsor	11
	5c	Role of study sponsor and funders, if any, in study design; collection, management, analysis, and interpretation of data; writing of the report; and the decision to submit the report for publication, including whether they will have ultimate authority over any of these activities	11
	5d	Composition, roles, and responsibilities of the coordinating centre, steering committee, endpoint adjudication committee, data management team, and other individuals or groups overseeing the trial, if applicable (see Item 21a for data monitoring committee)	5,6,9,10

For peer review only

Introduction

Background and rationale	6a	Description of research question and justification for undertaking the trial, including summary of relevant studies (published and unpublished) examining benefits and harms for each intervention	3____________
	6b	Explanation for choice of comparators	______________
Objectives	7	Specific objectives or hypotheses	4,5__________
Trial design	8	Description of trial design including type of trial (eg, parallel group, crossover, factorial, single group), allocation ratio, and framework (eg, superiority, equivalence, noninferiority, exploratory)	6____________

Methods: Participants, interventions, and outcomes

Study setting	9	Description of study settings (eg, community clinic, academic hospital) and list of countries where data will be collected. Reference to where list of study sites can be obtained	6____________
Eligibility criteria	10	Inclusion and exclusion criteria for participants. If applicable, eligibility criteria for study centres and individuals who will perform the interventions (eg, surgeons, psychotherapists)	7____________
Interventions	11a	Interventions for each group with sufficient detail to allow replication, including how and when they will be administered	5,6__________
	11b	Criteria for discontinuing or modifying allocated interventions for a given trial participant (eg, drug dose change in response to harms, participant request, or improving/worsening disease)	n/a___________
	11c	Strategies to improve adherence to intervention protocols, and any procedures for monitoring adherence (eg, drug tablet return, laboratory tests)	n/a___________
	11d	Relevant concomitant care and interventions that are permitted or prohibited during the trial	n/a___________
Outcomes	12	Primary, secondary, and other outcomes, including the specific measurement variable (eg, systolic blood pressure), analysis metric (eg, change from baseline, final value, time to event), method of aggregation (eg, median, proportion), and time point for each outcome. Explanation of the clinical relevance of chosen efficacy and harm outcomes is strongly recommended	8,9___________
Participant timeline	13	Time schedule of enrolment, interventions (including any run-ins and washouts), assessments, and visits for participants. A schematic diagram is highly recommended (see Figure)	8____________

For peer review only - http://bmjopen.bmj.com/site/about/guidelines.xhtml

Sample size	14	Estimated number of participants needed to achieve study objectives and how it was determined, including clinical and statistical assumptions supporting any sample size calculations	6____________
Recruitment	15	Strategies for achieving adequate participant enrolment to reach target sample size	6____________
**Methods: Assignment of interventions (for controlled trials)**			
Allocation:			
Sequence generation	16a	Method of generating the allocation sequence (eg, computer-generated random numbers), and list of any factors for stratification. To reduce predictability of a random sequence, details of any planned restriction (eg, blocking) should be provided in a separate document that is unavailable to those who enrol participants or assign interventions	n/a___________
Allocation concealment mechanism	16b	Mechanism of implementing the allocation sequence (eg, central telephone; sequentially numbered, opaque, sealed envelopes), describing any steps to conceal the sequence until interventions are assigned	n/a___________
Implementation	16c	Who will generate the allocation sequence, who will enrol participants, and who will assign participants to interventions	n/a___________
Blinding (masking)	17a	Who will be blinded after assignment to interventions (eg, trial participants, care providers, outcome assessors, data analysts), and how	n/a___________
	17b	If blinded, circumstances under which unblinding is permissible, and procedure for revealing a participant's allocated intervention during the trial	n/a___________
**Methods: Data collection, management, and analysis**			
Data collection methods	18a	Plans for assessment and collection of outcome, baseline, and other trial data, including any related processes to promote data quality (eg, duplicate measurements, training of assessors) and a description of study instruments (eg, questionnaires, laboratory tests) along with their reliability and validity, if known. Reference to where data collection forms can be found, if not in the protocol	9,10__________
	18b	Plans to promote participant retention and complete follow-up, including list of any outcome data to be collected for participants who discontinue or deviate from intervention protocols	9,10__________

Data management	19	Plans for data entry, coding, security, and storage, including any related processes to promote data quality (eg, double data entry; range checks for data values). Reference to where details of data management procedures can be found, if not in the protocol	9,10___________
Statistical methods	20a	Statistical methods for analysing primary and secondary outcomes. Reference to where other details of the statistical analysis plan can be found, if not in the protocol	9,10___________
	20b	Methods for any additional analyses (eg, subgroup and adjusted analyses)	9,10___________
	20c	Definition of analysis population relating to protocol non-adherence (eg, as randomised analysis), and any statistical methods to handle missing data (eg, multiple imputation)	_______________
**Methods: Monitoring**			
Data monitoring	21a	Composition of data monitoring committee (DMC); summary of its role and reporting structure; statement of whether it is independent from the sponsor and competing interests; and reference to where further details about its charter can be found, if not in the protocol. Alternatively, an explanation of why a DMC is not needed	nil____________
	21b	Description of any interim analyses and stopping guidelines, including who will have access to these interim results and make the final decision to terminate the trial	nil___________
Harms	22	Plans for collecting, assessing, reporting, and managing solicited and spontaneously reported adverse events and other unintended effects of trial interventions or trial conduct	6_____________
Auditing	23	Frequency and procedures for auditing trial conduct, if any, and whether the process will be independent from investigators and the sponsor	nil____________
**Ethics and dissemination**			
Research ethics approval	24	Plans for seeking research ethics committee/institutional review board (REC/IRB) approval	n/a___________
Protocol amendments	25	Plans for communicating important protocol modifications (eg, changes to eligibility criteria, outcomes, analyses) to relevant parties (eg, investigators, REC/IRBs, trial participants, trial registries, journals, regulators)	n/a___________

Consent or assent	26a	Who will obtain informed consent or assent from potential trial participants or authorised surrogates, and how (see Item 32)	6_____________
	26b	Additional consent provisions for collection and use of participant data and biological specimens in ancillary studies, if applicable	6_____________
Confidentiality	27	How personal information about potential and enrolled participants will be collected, shared, and maintained in order to protect confidentiality before, during, and after the trial	9,10__________
Declaration of interests	28	Financial and other competing interests for principal investigators for the overall trial and each study site	11____________
Access to data	29	Statement of who will have access to the final trial dataset, and disclosure of contractual agreements that limit such access for investigators	9,10__________
Ancillary and post-trial care	30	Provisions, if any, for ancillary and post-trial care, and for compensation to those who suffer harm from trial participation	n/a___________
Dissemination policy	31a	Plans for investigators and sponsor to communicate trial results to participants, healthcare professionals, the public, and other relevant groups (eg, via publication, reporting in results databases, or other data sharing arrangements), including any publication restrictions	2___________
	31b	Authorship eligibility guidelines and any intended use of professional writers	nil____________
	31c	Plans, if any, for granting public access to the full protocol, participant-level dataset, and statistical code	nil____________
**Appendices**			
Informed consent materials	32	Model consent form and other related documentation given to participants and authorised surrogates	supplementary_
Biological specimens	33	Plans for collection, laboratory evaluation, and storage of biological specimens for genetic or molecular analysis in the current trial and for future use in ancillary studies, if applicable	Supplementary_

*It is strongly recommended that this checklist be read in conjunction with the SPIRIT 2013 Explanation & Elaboration for important clarification on the items. Amendments to the protocol should be tracked and dated. The SPIRIT checklist is copyrighted by the SPIRIT Group under the Creative Commons "Attribution-NonCommercial-NoDerivs 3.0 Unported" license.

For peer review only - http://bmjopen.bmj.com/site/about/guidelines.xhtml

BMJ Open

DIALOR (DIgitAL cOaching for fRailty): Protocol for a single-arm mixed methods feasibility study of a digital health coaching intervention for older people with frailty in primary care.

Journal:	*BMJ Open*
Manuscript ID	bmjopen-2023-080480.R2
Article Type:	Protocol
Date Submitted by the Author:	29-Mar-2024

Complete List of Authors:	Sait, Matthew; Bournemouth University Faculty of Health and Social Sciences, Rehabilitation and Sport Sciences<br>Christie, Rachel; Bournemouth University, Rehabilitation and Sports Sciences<br>Cox, Chantel; Bournemouth University, Nursing Science<br>Board, Michele; Bournemouth University, Nursing Science<br>Thomas, Sarah; Bournemouth University , Faculty of Health and Social Sciences<br>O'Sullivan, Cheryl; NHS Dorset<br>Davies, Cheryl; Wessex Academic Health Science Network<br>Walker, Dawn-Marie; University of Southampton, Faculty of Health Sciences<br>Vassallo, Michael; University Hospitals Dorset NHS Foundation Trust, Older Person Medicine<br>Sadler, EA; University of Southampton, Faculty of Environmental and Life Sciences, School of Health Sciences<br>Allen-Pick, Mark; Bournemouth University, Rehabilitation and Sports Sciences<br>Moore, Patrick; The Adam Practice<br>Bradbury, Katherine; University of Southampton, Psychology<br>Murphy, Jane; Bournemouth University, Humans Sciences & Public Health

**Primary Subject Heading**:	Geriatric medicine
Secondary Subject Heading:	Health informatics, General practice / Family practice, Health services research, Qualitative research
Keywords:	Aging, Health informatics < BIOTECHNOLOGY & BIOINFORMATICS, Chronic Disease, Primary Health Care, Frailty

SCHOLARONE™
Manuscripts

**Title**	DIALOR (DIgitAL cOaching for fRailty): Protocol for a single-arm mixed methods feasibility study of a digital health coaching intervention for older people with frailty in primary care.
**Word count** (Excluding title page, abstract, references, figures, tables, or acknowledgements)	3689
**Key Words**	Aging, frailty, chronic disease, long-term conditions, health informatics, primary health care
**Authors' names**	Matthew Sait (0000-0003-0226-0351), Rachel Christie, Chantel Cox (0009-0000-7241-3179), Michele Board (0000-0002-6732-0151), Sarah Thomas (0000-0002-9501-9091), Cheryl Davies, Dawn-Marie Walker (0000-0003-2135-1363), Michael Vassallo, Euan Sadler (0000-0003-3827-224X), Cheryl O'Sullivan, Mark-Allen Pick, Patrick Moore (0000-0001-8279-6724), Katherine Bradbury (0000-0001-5513-7571), Jane Murphy (0000-0003-3531-5566)
**Author addresses, names, and positions**	Bournemouth University, 12 St Paul's Ln, BH8 8GP, Bournemouth, England Department of rehabilitation and sports science Matthew Sait Post-Graduate Researcher and Research Project Officer, Bournemouth University Rachel Christie research assistant, department of rehabilitation and sports science, Bournemouth University Chantel Cox senior lecturer in adult nursing department of nursing science, Bournemouth University Michele Board associate professor, Wessex AHSN Cheryl Davies senior programme manager innovation adoption, Bournemouth University Sarah Thomas deputy director Bournemouth university clinical research unit, University of Southampton Dawn-Marie Walker associate professor, University Hospitals Dorset Michael Vassallo consultant geriatrician, University of Southampton Euan Sadler associate professor, National Health Service (NHS) Dorset Cheryl O'sullivan advanced nurse practitioner, University Hospitals Dorset Mark-Allen Pick occupational therapist, The Adam Practice Patrick Moore GP Partner, University of Southampton Katherine Bradbury principal research fellow, Bournemouth University Jane Murphy deputy dean of research
**Corresponding author**	Correspondence to: Sait, Matthew Ian on saitm@bournemouth.ac.uk

For peer review only - http://bmjopen.bmj.com/site/about/guidelines.xhtml

Abstract (300 words)

Background

Multi-domain interventions in older adults offer the best opportunity to prevent, delay, or reverse existing symptoms in the earlier stages of frailty and improve independence but can be costly, and difficult to deliver at scale. However, digital health interventions enable personalised care and empowerment through self-management of long-term conditions, used at any time and when combined with health coaching offer potential to enhance wellbeing and facilitate achievement of health-related goals. We aim to evaluate the feasibility and acceptability of a digital health platform for long-term disease management combined with health coaching for people living with mild-moderate frailty, targeting self-identified goals – activity, nutrition, mood, enhancing social engagement and well-being.

Methods and analysis

This is a non-randomised feasibility, single group, pre-test/post-test study, using qualitative and quantitative methods. The digital health coaching intervention (DIALOR - DIgitAL cOaching for fRailty) has been developed for implementation to older adults, aged 65 years or older with mild to moderate frailty and diagnosis of one or more long-term health conditions in the community. Participants will receive 12 weeks of health coaching and have access to a mobile health platform for 6 months. The primary outcome measure is the acceptability and feasibility of DIALOR along with a range of secondary outcome measures (including frailty, functioning measures, quality of life, social engagement, diet quality and self-reported indicators) collected at baseline and at 6 months. The findings will inform whether a wider effectiveness trial is feasible and if so, how it should be designed.

Ethics and Dissemination

Ethical approval has been granted by the Southeast Scotland Research Ethics Committee 02 (reference: 22/SS/0064). Research findings will be disseminated in a range of different ways to engage different audiences, including publishing in open-access peer-reviewed journals, conference presentations, social media, dissemination workshop with patients, carers, and healthcare professionals and on institution websites.

Article Summary

Strengths and limitations of this study

- A strength of the study design is using a mixed methods approach with qualitative understanding of the implementation process and quantitative measures to assess the effects on health outcomes and whether the trial is feasible.
- There is strong involvement of stakeholders and people with the lived experience to develop and guide the study.

For peer review only - http://bmjopen.bmj.com/site/about/guidelines.xhtml

- Potential limitations are as a feasibility study, it will assess a pre- and post-test intervention with non-randomisation of sample and to sufficiently engage hard-to reach target groups including those with poor digital literacy and low socio-economic status, impacting representation of the participant group and generalisability of the findings.

Introduction

Frailty is a multidimensional syndrome characterised by cumulative loss of reserves and accumulation of deficits (e.g., energy, physical ability, cognition, social and psychological aspects) that give rise to vulnerability and risk of adverse outcomes with advancing age (1). It is not an inevitable consequence of ageing but older adults with frailty have increased risk of poor health, including falls, fractures and functional impairment, hospitalisation, need for nursing home care, and increased mortality (2). Older people with frailty experience impairment in their activities of daily living and often report significant reduction in their quality of life (3). Furthermore, studies have shown that frailty may be related to decreased mood (4) and may be shaped by social factors such as different levels of social support or living alone (5). Frailty affects about 12% of people aged ≥ 65 years worldwide (6) and 14% of older people in the United Kingdom (UK) (7), and even higher numbers of individuals in the early stages of frailty, ranging from 19-53% (8). Frailty is an ever-present burden amongst international populations, which has been exacerbated by the impact of the Covid-19 pandemic (9) and the resultant deconditioning (10).

Whilst moderate to severe frailty has a higher risk of physical health declines, mild-moderate frailty with some loss of physiological reserve is considered to be potentially reversible (11) to a robust or stable state (12). Mild frailty is conceptualised as an intermediate stage on the Rockwood Clinical Frailty Scale (13), whereby older people experience some loss of physiological reserves but can usually recover after a negative stressor event, such as an infection or fall. This would indicate that interventions targeting earlier stages of frailty are more likely to reverse frailty decline. Moreover, there is evidence to suggest that interventions, particularly multi-domain interventions in older adults including mobility, strength, balance, nutrition, physical activity, and social support offer the best opportunity to prevent, delay, or reverse existing symptoms of frailty and improve independence (14, 15, 16, 17). Most multi-component interventions incorporate involvement from a multidisciplinary team of health and social care professionals meaning they can be costly, and difficult to deliver at scale (18).

Digital health interventions (DHIs) enable personalised care and empowerment through self-management of a long-term condition, which can be used at any time, and may offer more cost-effective health care delivery (19). DHIs have been shown to be effective at achieving successful remote monitoring and management of COPD amongst older populations (20), suggesting that DHIs may be a feasible method of reaching remote communities. Studies show that digital approaches for lifestyle modifications in other long-term conditions such as Type 2 diabetes, offer effective and scalable options when face-to face or in-person programmes are not accessible or feasible, especially when offered with health coaching (21, 22).

Health Coaching was originally described as the practice of health education and health promotion within a coaching context, to enhance the wellbeing of individuals and to facilitate achievement of health-related goals (23). More recently, HC has been defined as helping people gain and use the knowledge, skills, and confidence to become active participants in their care so that they can reach

For peer review only - http://bmjopen.bmj.com/site/about/guidelines.xhtml

their self-identified health and wellbeing goals (24). In the last decade, HC has become increasingly embedded within Integrated Care Services. However, the evidence base demonstrating the effectiveness of HC in the UK is limited. For example, a systematic review and meta-analysis assessing the impact of HC on Type 2 diabetes found that HC was effective in achieving optimal glycaemic levels via self-management strategies (25). In the UK, health coaches working within primary care can be an established clinical team member (e.g., registered nurses, physician associates, physiotherapists, paramedics, occupational therapists, and psychologists) or can be unlicensed health workers (26). Furthermore, research is emerging to show that HC may offer a promising approach to support effective lifestyle interventions for managing chronic conditions such as asthma, diabetes, cardiovascular disease, and mental illness on an international stage (27).

Current Integrated Care Systems in the UK have also implemented both DHIs via mHealth and HC to improve self-management of their long-term conditions (LTC), amongst older people living with COPD, diabetes, and cardiovascular disease (28). However, there remains a distinct lack of evidence which demonstrates the effectiveness of HC for older adults who have at least two chronic conditions (multimorbidity), which is thought to affect up to 50% of older people (aged over 65 years) (29, 30). Further, the impact of HC for older adults with a diagnosis of frailty, is unknown, making a single disease focused model of healthcare unsuitable to provide effective management (31). Therefore, targeted behaviour change interventions using DHIs such as mHealth, in conjunction with HC, have potential to offer a proactive, and wide-reaching approach to prevent frailty decline alongside the management of other diagnosed co-morbidities and improve health and wellbeing outcomes.

This study aims to address this gap by co-designing and implementing a multicomponent intervention for people living with mild-moderate frailty. The DIALOR intervention will include two key components (Figure 1);

1) Digital health coaching
    - This will include digital health coaching sessions with a qualified health coach. An online platform (Zoom/Accurx/Microsoft Teams) will be used to deliver the digital HC intervention.
2) Mobile health platform
    - A suite of web-based platforms offering tailored clinical advice and support and self-management resources for management of chronic obstructive pulmonary disease (COPD), Asthma, cardiovascular disease (CVD) and type 2 diabetes (details of the online platform can be accessed via https://mymhealth.com/). The platforms can be accessed by any electronic device (e.g., smartphone, tablet, or television) that has an active internet connection.

The primary objectives of this study are to:

- Explore barriers and facilitators of the digital intervention from the perspectives of health coaches and healthcare professionals (HCPs) and determine the essential and desirable features of the intervention (wants and needs; preferred outcomes) to identify what functions will be most useful for the target population.

For peer review only - http://bmjopen.bmj.com/site/about/guidelines.xhtml

- Explore how HC can provide support for the management of mild-moderate frailty and long-term conditions from the perspectives of older people with mild-moderate frailty, carers, HCPs, and health coaches.
- Determine the acceptability and feasibility of the intervention for older adults with mild/moderate frailty and another long-term condition referred to access health coaching services and willing to engage with the mobile health platform.

The secondary objectives of this study are:

- Explore the factors which inhibit or facilitate implementation including contextual factors, recruitment processes, resources needed, potential mechanisms and pathways to impact.
- Explore the acceptability and suitability of outcomes measures (to inform sample size estimation and determine the primary outcome measure) for a future randomised controlled trial (RCT) of the intervention (should it be feasible).
- Measure key domains for secondary outcome measures including participants who are able to self-report measures.

Figure 1: Overview of the DIALOR intervention and its functionalities

Methods and Analysis

Intervention Development

Over a 9-month period, intervention development will use guiding principles (32) to inform co-design objectives of a logic model (Figure 2) and ensure the intervention is acceptable, feasible, engaging, and easy to implement. A systematic review and narrative synthesis will also guide the development of key features our intervention requires to meet user needs and best practice guidelines.

Behavioural analysis (33) will triangulate evidence from the i. the narrative synthesis, ii. consultation with two Stakeholder Engagement Groups (SEG) including HCPs, health coaches, commissioners of service and patient and iii. public involvement (PPI) advisors, as well as iv. a separate PPI group with lay advisors to identify barriers in each domain - activity/mobility, nutrition, social engagement, and psychological well-being. Intervention components addressing each barrier will be theoretically mapped and characterised using the Behaviour Change Wheel (BCW) (34), the Theoretical Domains Framework (TDF) (35) and Normalisation Process Theory (NPT). NPT is the process through which complex, new ways of working become embedded into standard practice (36). We will use the Behaviour Change Techniques Taxonomy Version 1 (BCTT v1) (37) to code the behaviour change techniques delivering the identified intervention functions. This will provide a clear description of the intervention and factors which may influence its implementation and will be summarised in a behavioural analysis table. We will then convene another SEG meeting to discuss and refine the proposed logic model.

To identify challenges/enabling factors for delivery/implementation and intervention modifications needed to maximise engagement and behaviour change, we will conduct individual interviews with people with mild to moderate frailty (and their carers where appropriate) (n=20) and focus groups (n=4) with HCPs and health coaches (n=20) (Figure 3). We will undertake purposive sampling to select older participants (following ethics protocol for consent), up to 20 semi-structured interviews or until data saturation is reached, in accordance with robust methods in qualitative research (38). These will be conducted face-to-face, and audio recorded using dictaphones, otherwise they will be conducted and recorded through Microsoft Teams or Zoom. Final in-house testing of the

For peer review only - http://bmjopen.bmj.com/site/about/guidelines.xhtml

intervention will be undertaken before the commencement of the study. We will use the TIDieR checklist (39) for intervention description which will report relevant outcomes to inform further planning for a future trial.

Study Design

This feasibility study is a non-randomised, single group, pre-test/post-test study, using qualitative and quantitative methods. This protocol was developed in accordance with the Standard Protocol Items: Recommendations for Intervention Trials (SPIRIT) reporting guidelines (40).

This will be a mixed methods prospective cohort study conducted within primary care settings in Southwest England. Firstly, online group training of the intervention will be provided for health coaches by the research team, with top-up training and support as necessary throughout. A training manual will also incorporate frailty awareness, and the CARE (Congratulate, Ask, Reassure, Encourage) model approach (41, 42). Intervention delivery will be tailored to each patient, with goals and strategies to help achieve them developed in conjunction with the health coach.

Study setting

The study will work with GP practices in Primary Care Networks (PCN) in Southwest England to identify patients already using the mobile health platform. GP practices will be eligible to take part if they are currently referring older people with mild to moderate frailty to access HC/social prescribing services and willing to test new digital approaches.

Participant recruitment

Participant recruitment will be conducted within primary care services, at individual participating GP surgeries. Up to 50 older people with mild-moderate frailty will be recruited in line with previous sample size recommendations for feasibility studies (43). Eligibility criteria for older adults with mild to moderate frailty to participate in the study are outlined in Table 1. The clinical care team from participating GP surgeries will identify eligible participants through existing medical records.

Three strategies will be utilised to recruit patients i) via text message invitation to eligible patients which provides a link to a website with further information about the study, participant information sheet and contact information for the research team. Potential participants will be followed up by the research team to provide an opportunity to answer questions, confirm eligibility and obtain informed consent to participate, ii) by a member of the research team approaching eligible participants at the GP surgery, signposted by the clinical team and iii) via leaflets provided to eligible patients by the clinical team.

Table 1: Inclusion and exclusion criteria

Inclusion criteria
• Aged ≥ 65 years<br>• Scoring as mild or moderately frail using the Electronic Frailty Index<br>• Diagnosis of 1 or more long-term conditions including COPD, Type 2 diabetes, heart disease or asthma<br>• Living in their own homes<br>• Capacity to consent to participate<br>• Able to communicate fluently enough in English to take part in the study<br>• Able to recall their experiences sufficiently to engage with a health coach<br>• Self-reported ability to use digital technology independently or with support
**Exclusion criteria**
• Nursing home resident or on active waiting list for a place<br>• Individuals who have a terminal illness and are being managed for end-of-life needs<br>• Lacking capacity to consent (e.g., advanced dementia)

For peer review only - http://bmjopen.bmj.com/site/about/guidelines.xhtml

Figure 2: Proposed logic model

Figure 3: Process flow of study design

Digital Support for patients

Patients with low digital literacy will be supported by the research team to access the digital HC and mobile health platforms. They will be offered a training manual, developed by the research team, and co-produced alongside the SEG and PPI group alongside digital support leaflets and guidance on keeping safe online. To reduce the risk of digital inequity, internet enabled tablets may be provided to individuals who lack a device, for the length of the study. Internet and data packages (via local council schemes) will be provided if the participant lacks access to an established internet connection. Digital support will be offered throughout the study via local council services (volunteer digital upskilling clinics embedded within public spaces e.g., GP surgeries and council libraries) and the research team.

Outcome measures

Primary outcomes

The primary outcome measures are feasibility and acceptability of the DIALOR intervention. Feasibility will be determined from the number of health coaches trained, number of participants recruited, retention and adherence to the intervention as well as any adverse events. To determine the acceptability of the intervention and to explore barriers and enablers to the implementation of the intervention, semi-structured interviews will be conducted with patients with mild-moderate frailty (and their carers where appropriate) (n= 20), HCPs and health coaches (n= 20) who participated in the study.

The interviews will consist of open-ended questions to explore participant views of the intervention, barriers, and facilitators to inform the design of further studies. To understand the factors influencing implementation, the interview schedule will be underpinned by normalisation process theory (NPT) (44). NPT is an implementation theory providing a framework to identify and explain important elements of the implementation process. As such, it informs an understanding of the social processes through which new or practice can be implemented, embedded, and integrated into care settings.

Secondary outcomes

The secondary outcomes will include the measurement of functioning, quality of life, physical activity levels, frailty severity, diet quality, and engagement using validated outcome measures. Physical functioning, a key outcome in frailty trials (45), will be measured using the Modified Barthel Index (MBI) (46) and Lawton-Brody Instrumental Activities of Daily Living (IADL) (47). The MBI will measure older adults' functional ability using 10 items, with a higher score reflecting higher ability to function independently. The MBI has been found to be of reasonable reliability when used amongst older adults (48). The IADL will measure more complex activities of daily living using 8 key domains. The International Physical Activity Questionnaire short form (IPAQ-SF) will measure older adults activity levels in the last 7 days (49). The evidence supporting the IPAQ-SF as an indicator of relative

For peer review only - http://bmjopen.bmj.com/site/about/guidelines.xhtml

or absolute physical activity is weak (50). However, it is a validated generalised outcome measure for older adult populations (65 years or over) (51).

Quality of life is a core outcome in frailty trials (52) and will be measured using the EuroQol-5 dimensions-5 levels (EQ-5D-5L) questionnaire which includes 5 health areas (mobility, self-care, usual activities, pain/discomfort, and anxiety/depression). These 5 health areas are assessed with 5 main response levels (no problems, slight, moderate, severe, or unable), along with a visual analogue scale (53).

Frailty severity will be measured using the Groningen Frailty Indicator (GFI) (54). The GFI is a multidimensional screening instrument consisting of 15 self-report items. It is a feasible and widely used screening instrument for identifying frail older adults (55, 56). Higher GFI scores indicate higher frailty levels and an increased need for integrated care (57).

The Short Form Food Frequency Questionnaire (SFFFQ) will measure dietary quality. The SFFQ asks about foods which respondents might have consumed, on average, over a set time period (3 months) and is found to be an effective instrument for assessing diet quality in large scale studies with UK adult populations (58).

Social engagement will be measured using the Lubben Social Network Scale (LSNS-6) and mental wellbeing will be measured using the Warwick-Edinburgh Mental Well-Being Scale (WEMWBS) Self-reported indicators such as height, weight, BMI (Body Mass Index), number of falls (per year), goal attainment, alcohol intake and smoking habits (if applicable) will be measured at baseline (pre-intervention), throughout the study during digital HC intervention, and at 6 months (post-intervention).

Data will be collected from the mobile health platform which will be recorded automatically by inbuilt tracking software, including usage patterns from number of logins, and length of time that older people and their carers use the platform. These data will be collected at 6 months for each patient participant (post-intervention).

Data Analysis

Quantitative data analysis

Data will be entered into a secure database for analyses. Statistical analyses will be conducted using statistical software SPSS (V.28.0). Descriptive statistics: median (IQR); mean (SD); number (%) and confidence interval – will be used to analyse the number of volunteers recruited and retained, as well as participants' adherence to the intervention and pre-post intervention outcome measures to assess the feasibility of delivering the intervention. Pre- and post-analyses at 6 months, will be conducted to determine if the intervention had an impact on the lifestyle management of frailty measured by functioning (MBI, IADL), social engagement (LSNS-6), mental wellbeing (WEMWBS) quality of life (EQ-5D-5L), physical activity (IPAQ-SF), frailty indicator (GFI), diet quality (SFFFQ) and self-reported indicators.

Qualitative data analysis

For peer review only - http://bmjopen.bmj.com/site/about/guidelines.xhtml

Data collected from all the interviews and focus groups will be transcribed verbatim and analysed using thematic analysis (TA). TA is a commonly used method for identifying, analysing, and reporting patterns or themes within data (59). There are six key phases: Phase 1 – familiarisation with the data, Phase 2 – initial code generation, Phase 3 – searching for and generating themes, Phase 4 – reviewing themes, Phase 5 – defining and naming themes and Phase 6 – producing the report. Analysis of qualitative data will be conducted using Microsoft word, and or alternative software such as NVivo (V.12). RC will analyse codes using NPT (44) and the Behaviour Change Technique Taxonomy (37) to focus on the social processes shaping the implementation process from making sense of the intervention to engaging in it (individually and collectively) and embedding this (normalising) in everyday practice (or people's everyday lives) and to identify the facilitators and barriers present throughout the implementation process. Themes will be developed, from the codes, to reflect the lived experience and views of patient participants, health coaches, and HCPs regarding the digital HC and mobile health platform intervention. MS will code 25% of interviews and focus groups separately to develop, discuss, and agree themes with RC. Finally, members of the core research team (ES, MB, CC, JM) will be consulted to develop, discuss, and agree themes via an iterative process.

Patient and Public Involvement

Public/patient involvement and engagement (PPI) has been and will continue to be part of all stages of the research from developing the proposal to providing feedback on preliminary findings, to dissemination and impact, and input for tasks such as finalising interview/focus group topic guides. For the development of this programme of research, we consulted with seven older adults and family carers who helped inform the proposal. They thought that this was an important topic and liked the HC approach to support digital engagement. We have convened a PPI advisory group (6-10 participants, aged 60+ years, meeting 3 to 4 times throughout the project). A PPI representative as a co-researcher leads the PPI group and attends the project advisory group. The group represents the views of people from different socio-economic backgrounds including those who do and do not have access to various online and digital technologies.

PPI meetings will be face-to-face, and advisors will be offered reimbursement aligning with National Institution for Health and Care Research (NIHR) payment guidance. Advisors will have the opportunity to contribute via email and phone between meetings. Advisors will be sent materials prior to meetings and able to discuss matters arising from meetings afterwards. Advisors will be provided with meeting summaries and feedback on how their involvement informed the research project. A PPI plan will be developed and shaped by PPI and results/activities will be shared with the project advisory group. Monitoring and reporting will use the GRIPP checklist (60).

Ethics and dissemination

The DIALOR project is sponsored by Bournemouth University. Ethical approval has been granted by the Southeast Scotland Research Ethics Committee 02 (reference: 22/SS/0064). Data collected during the study will be pseudonymised by a member of the research team (RC) to ensure removal of identifying information from research data prior to data analysis. Data will be stored on Bournemouth University servers, and identification data will be stored in encrypted files in a separate, secure location. Data transfer will be undertaken through secure, encrypted emails or Bournemouth University's secure file transfer system (BU Transfer). We will publish results in peer-

For peer review only - http://bmjopen.bmj.com/site/about/guidelines.xhtml

reviewed open access journals. A lay summary of the final study report will be accessible on the project website: (https://www.bournemouth.ac.uk/research/projects/dialor-digital-coaching-frailty). We will also seek to present our findings at conferences, workshops for patients, write lay articles for dissemination to the public, and share the findings through our networks across health and social care.

Status

The trial commenced September 2023 and is expected to be completed by September 2024.

Acknowledgements

The authors thank study participants and patient advisers for their input. The authors wish to thank GP director Simone Yule for helping to implement the study within local primary care services. The authors would like to thank colleagues from Dorset Help & Care charity and my mhealth for helping to implement the study intervention and imbed this seamlessly within primary care services.

Author Contributions

The study concept and design was conceived by JM. MS, RC, ES, CC, and MB assisted in refining the study questionnaires and study design. MS, RC, and MAP will be responsible for data collection. Analyses will be conducted by MS, RC, ES, DMW, CS, KB, CC, MB, JM, and MAP. MS prepared the first draft of the manuscript. All authors critically revised the manuscript and approved the submitted version. The corresponding author attests that all listed authors meet authorship criteria and that no others meeting the criteria have been omitted.

Funding

This research was support by NIHR Applied Research Collaborative Wessex, grant number (ARC 001).

Disclaimer

Provenance and peer review not commissioned; externally peer reviewed.

Competing interests

None declared.

Copyright/license

The Corresponding Author has the right to grant on behalf of all authors and does grant on behalf of all authors, a worldwide licence to the Publishers and its licensees in perpetuity, in all forms, formats and media (whether known now or created in the future), to i) publish, reproduce, distribute, display and store the Contribution, ii) translate the Contribution into other languages, create adaptations, reprints, include within collections and create summaries, extracts and/or, abstracts of the Contribution, iii) create any other derivative work(s) based on the Contribution, iv) to exploit all subsidiary rights in the Contribution, v) the inclusion of electronic links from the Contribution to third party material where-ever it may be located; and, vi) licence any third party to do any or all of the above.

For peer review only - http://bmjopen.bmj.com/site/about/guidelines.xhtml

References

1. Rockwood, K., Song, X., MacKnight, c., Bergman, H., Hogan, D., McDowell, I., Mitnitski, A., 2005. A global clinical measure of fitness and frailty in elderly people, *Canadian Medical Association Journal*, 173 (5), 489-495.
2. Clegg, A., Young, J., Iliffe, S., Rikkert, M., Rockwood, K., 2013. Frailty in elderly people, *Lancet*, 381 (9868), 1-25.
3. Langlois, F., Vu, T., Chasse, K., Dupuis, G., Kergoat, M J., Bherer, L., 2012. Benefits of physical exercise training on cognition and quality of life in frail older adults, The *Journals of Gerontology, Series B, Psychological Sciences and Social Sciences,* 68 (3), 400-404.
4. Collard, R., Comijs, H., Naarding, P., Penninx, B., Milaneschi, Y., Ferruci, L., Voshaar, R., 2015. Frailty as a Predictor of the Incidence and Course of Depressed Mood, *Journal of the American Medical Directors Association*, 16 (6), 509-514.
5. Robertson, D., Savva, G., Kenny, R., 2013. Frailty and cognitive impairment – A review of the causal mechanisms, *Ageing Research Reviews*, 12 (4), 840-851.
6. O'Caoimh, R., Sezgin, D., O'Donovan, M,R., Molloy, D,W., Clegg, A., Rockwood, K., 2021. Prevalence of frailty in 62 countries across the world: a systematic review and meta-analysis of population-level studies, Age Ageing, 50 (1), 96–104.
7. Gale, C., Cooper, C., Deary I., Sayer A., 2014. Psychological well-being and incident frailty in men and women: the English Longitudinal Study of Ageing, *Psychological Medicine*, 44 (4), 697-706.
8. Collard, R., Boter, H., Schoevers, R., Voshaar, R., 2012. Prevalence of Frailty in Community-Dwelling Older Persons: A Systematic Review, *Journal of the American Geriatrics Society*, 60 (8), 1487-1492.
9. Hussien, H., Nastasa, A., Apetrii, M., Nistor, I., Petrovic, M., Covic, A., 2021. Different aspects of frailty and COVID-19: points to consider in the current pandemic and future ones, *BMC Geriatrics*, 21 (389), 1-11.
10. Steinman, M., Perry, L., Perissinotto, C., 2020. Meeting the Care Needs of Older Adults Isolate at Home During the COVID-19 Pandemic, *JMA Internal Medicine*, 180 (6), 819-820.
11. Ng, T., Feng, L., Nyunt, M., Feng, L., Niti, M., Tan, B., Chan, G., Khoo, S., Chan, S., Yap, P., Yap, K., 2015. Nutritional, Physical, Cognitive, and Combination Interventions and Frailty Reversal Among Older Adults: A Randomized Controlled Trial, *The American Journal of Medicine*, 128 (11), 1225-1236.
12. Gill, T., Gahbauer, E., Allore, H., Han L., 2006. Transitions between frailty states among community-living older persons, *Archives of Internal Medicine*, 166 (4), 418-423.
13. Rockwood, K., Theou, O., Mitnitski, A., 2015. What are frailty instruments for? *Age and Ageing*, 44, 545-547.
14. Cameron, I., Fairhall, N., John, B., Lockwood, K., Monaghan, N., Sherrington, C., 2017. A multifactorial interdisciplinary intervention in pre-frail older people: randomised trial, *Innov Aging*, 1 (196)
15. Walters, K., Frost, R., Kharicha, K., Avgerinou, C., Gardner, B., Ricciardi, F., 2017. Home-based health promotion for older people with mild frailty: the Home Health intervention development and feasibility RCT, *Health Technol Assess*, 21 (73), 1–128.

For peer review only - http://bmjopen.bmj.com/site/about/guidelines.xhtml

16. Serra-Prat, M., Sist, X., Domenich, R., Jurado, L., Saiz, A., Roces, A., 2017. Effectiveness of an intervention to prevent frailty in pre-frail community-dwelling older people consulting in primary care: a randomised controlled trial, Age Ageing, 46 (3), 401–7.
17. Yu, R., Tong, C., Ho, F., Woo, J., 2020. Effects of a Multicomponent Frailty Prevention Program in Prefrail Community-Dwelling Older Persons: A Randomized Controlled Trial. J Am Med Dir Assoc, 21 (2), e1- e10.
18. Chen, Z., Ding, Z., Chen, C., Sun, Y., Jiang, Y., Liu, F., Wang, S., 2021. Effectiveness of comprehensive geriatric assessment intervention on quality of life, caregiver burden and length of hospital stay: a systematic review and meta-analysis of randomised controlled trials, *BMC Geriatrics*, 21(1), 377.
19. Gomes, M., Murray, E., Raftery, J., 2022. Economic Evaluation of Digital Health Interventions: Methodological Issues and Recommendations for Practice, *PharmacoEconomics*, 40, 367-378.
20. Velardo, C., Shah, S., Gibson, O., Clifford, G., Heneghan, C., Rutter, H., Farmer, A., Tarassenko, L., 2017. Digital health system for personalised COPD long-term management, *BMC Medical Informatics and Decision Making*, 17 (19), 1-13.
21. Smith, A., Crippa, A., Woodcock, J., Brage, S., 2016. Physical activity and incident type 2 diabetes mellitus: a systematic review and dose-response meta-analysis of prospective cohort studies, *diabetologia*, 59 (12), 2527-2545.
22. Castro Sweet, C., Chiguluri, V., Gumpina, R., Abbott, P., Madero, E., Payne, M., Happe, L., Matanich, R., Renda, A., Prewitt, T., 2018. Outcomes of a Digital Health Program With Human Coaching for Diabetes Risk Reduction in a Medicare Population, *Journal of Aging and Health*, 30 (5), 692-710.
23. Palmer, S., Cooper, C., Thomas, K., 2003. Creating a Balance: Managing Stress. London: British Library.
24. NHS England 2019. Universal Personalised Care: Implementing the Comprehensive Model.
25. Pirbaglou, M., Katz, J., Motamed, M., Pludwinski, S., Walker, K., Ritvo, P., 2018. Personal Health Coaching as a Type 2 Diabetes Mellitus Self-Management Strategy: A Systematic Review and Meta-Analysis of Randomized Controlled Trials, *American Journal of Health Promotion*, 32 (7), 1613-1626.
26. Gastala, N., Wingrove, P., Gaglioti, A., Liaw, W., Bazemore, A., 2018. The Growing Trend of Health Coaches in Team Based Primary Care Training, *Family medicine*, 50 (7), 526-530.
27. Yang, J., Bauer, B., Lindeen, S., Perlman, A., Dabrh, A., Boehmer, K., Salinas, M., Cutshall, S., 2020. Current trends in health coaching for chronic conditions., *Open Medicine*, 99 (30), 1-6.
28. McShane, M. and Kirkham, K. (2020). Making it personal – population health management and the NHS", *Journal of Integrated Care*, 28 (3), 243-252.
29. Barnett, K., Mercer, SW., Norbury, M., Watt, G., Wyke, S., Guthrie, B., 2012. Epidemiology of multimorbidity and implications for health care, research, and medical education: a cross-sectional study. *Lancet 2012*; 380 (9836), 37–43.
30. Salive, ME., 2013. Multimorbidity in older adults. *Epidemiol Rev*, 35, 75–83.
31. Kingston, A., Robinson, L., Booth, H., Knapp, M., Jagger, C., 2018. Projections of multi-morbidity in the older population in England to 2035: estimates from the Population Ageing and Care Simulation (PACSim) model, *Age and Ageing*, 47 (3), 374-380.
32. Yardley, L., Morrison, L., Bradbury, K., Muller, I., 2015. The person-based approach to intervention development: application to digital health-related behavior change interventions, *Journal of medical Internet research*, 17 (1), 1-15.

For peer review only - http://bmjopen.bmj.com/site/about/guidelines.xhtml

33. Band, R., Bradbury, K., Morton, K., May, C., Michie, S., Mair, F., Murray, E., McManus R., Little, P., Yardley, L., 2017. Intervention planning for a digital intervention for self-management of hypertension: a theory-, evidence- and person-based approach, *Implementation Science*, 12 (25), 1-13.
34. Michie, S., van Stralen, M.M., West, R., 2011. The behaviour change wheel: A new method for characterising and designing behaviour change interventions. *Implementation Science,* 42 (6), 1-11.
35. Cane, J., O'Connor, D., Michie, S., 2012. Validation of the theoretical domains framework for use in behaviour change and implementation research. *Implementation Science*, 37 (7), 1-17.
36. May, C., Cummings, A., Girling, M., Bracher, M., Mair, F., May, C., Murray, E., Myall, M., Rapley, T., Finch, T., 2018. Using Normalization Process Theory in feasibility studies and process evaluations of complex healthcare interventions: a systematic review, *Implementation Science*, 13 (80), 1-27.
37. Michie, S., Richardson, M., Johnston, M., Abraham, C., Francis, J., Hardeman, W., Eccles, M., Cane, J., Wood, C., 2013. The behaviour change taxonomy (v1) of 93 hierarchically clustered techniques: building an international consensus for the reporting of behaviour change interventions, *Annals of Behavioural Medicine*, 46 (1), 81-95.
38. Francis, J., Johnston, M., Robertson, C., Glidewell, L., Entwistle, V., Eccles, M., Grimshaw, J., 2010. What is an adequate sample size? Operationalising data saturation for theory-based interview studies, *Psychology & Health*, 25 (10), 1229-1245.
39. Hoffman, T., Glasziou, P., Milne, R., Moher, D., Barbour, V., Johnston, M., Lamb, S., Dixon-Woods, M., Wyatt, J., 2014. Better reporting of interventions: a template for intervention description and replication (TIDieR) checklist and guide, *BMJ*, 348, 1-12.
40. Chan, A, W., Tetzlaff, J., Gøtzsche, P., Altman, D., Mann, H., Berlin, J., Dickersin, K., Hróbjartsson, A., Schulz, K., Parulekar, W., Krleža-Jerić, K., Laupacis, A., Moher, D., 2013. SPIRIT 2013 statement: defining standard protocol items for clinical trials, *Annals of Internal Medicine*, 158 (3), 200-207.
41. Bradbury, K., Morton, K., Band, R., May, C., McManus, R., Little, P., Yardley, L., 2017. Understanding how primary care practitioners perceive an online intervention for the management of hypertension, *BMC Medical Informatics and Decision* Making, 17 (5), 1-13.
42. Smith, E., Bradbury, K., Scott, L., Steele, M., Little, P., Yardley, L., 2017. Providing online weight management in primary care: a mixed methods process evaluation of healthcare practitioners' experiences of using and supporting patients using POWeR+, *Implementation Science*, 12 (69), 1-13.
43. Billingham, S., Whitehead, A., Julious, S., 2013. An audit of sample sizes for pilot and feasibility trials being undertaken in the United Kingdom registered in the United Kingdom Clinical Research Network database, *BMC Medical Research and Methodology*, 13 (104), 1-6.
44. May, C., Mair, F., Finch, T., MacFarlane, A., Dowrick, C., Treweek, S., Rapley, T., Ballini, L., Ong, B., Rogers, A., Murray, E., Elwyn, G., Légaré, f., Gunn, J., Montori, V., 2009. Development of a theory of implementation and integration: Normalization Process Theory, *Implementation Science*, 4 (29), 1-9.
45. Tappenden, P., Campbell, F., Rawdin, A., Wong, R., Kalita, N., 2012. The clinical effectiveness and cost-effectiveness of home-based, nurse-led health promotion for older people: a systematic review, *Health Technology Assessment*, 16 (20), 1-72.

For peer review only - http://bmjopen.bmj.com/site/about/guidelines.xhtml

46. Wade, DT., and Collin, C., 1988. The Barthel ADL Index: a standard measure of physical disability, *International Disability Study*, 10, 64-67.
47. Lawton, M.P., and Brody, E.M, 1969. Assessment of older people: Self-maintaining and instrumental activities of daily living. The Gerontologist, 9 (3), 179-186.
48. Hopman-Rock, M., Hirtum, H., Vreede, P., Freiberger, E., 2019. Activities of daily living in older community-dwelling persons: a systematic review of psychometric properties of instruments, *Aging Clinical and Experimental Research*, 31, 917-925.
49. Hagstromer, M., Oja, P., Sjostrom, M., 2006. The international physical activity questionnaire (IPAQ): a study of concurrent and construct validity. *Public Health Nutrition*, 9 (6), 755-762.
50. Lee, P., Macfarlane, D., Lam, T., Stewart, S., 2011. Validity of the international physical activity questionnaire short form (IPAQ-SF): A systematic review. *International Journal of Behavioural Nutrition and Physical Activity*, 8 (115), 1-11.
51. Hurtig-Wennlöf, A., Hagströmer, M., Olsson, LA., 2010. The International Physical Activity Questionnaire modified for the elderly: aspects of validity and feasibility. *Public Health Nutr*, 13, 1847–54.
52. Prorok, J., Williamson, P., Shea, B., Rolfson, D., Manas, L., Cesari, M., Kim, P., Muscedere, J., 2022. An international Delphi consensus process to determine a common data element and core outcome set for frailty: FOCUS (The Frailty Outcomes Consensus Project), *BMC Geriatrics*, 22 (284), 1-9.
53. The EuroQoL Group. EuroQol--a new facility for the measurement of health-related quality of life. Health Policy 1990;16:199-208.
54. Steverink, N., 2001. Measuring frailty: developing and testing the GFI (Groningen frailty Indicator). *The Gerontologist*, 41, 236.
55. Metzelthin S, Daniëls R, Van Rossum E, De Witte L, van den Heuvel W, Kempen G., 2010. The psychometric properties of three self-report screening instruments for identifying frail older people in the community. *BMC Publ Health*, 10 (1), 176–183.
56. Peters LL, Boter H, Buskens E, Slaets JPJ., 2012. Measurement properties of the groningen frailty indicator in home-dwelling and institutionalized elderly people, *J Am Med Dir Assoc*, 13, 546–551.
57. Slaets, J,P., 2006. Vulnerability in the elderly: frailty. *Med Clin North Am*, 90 (4), 593–601.
58. Cleghorn, C., Harrison, R., Ransley, J., Wilikinson, S., Thomas, J., Cade, J., 2016. Can a dietary quality score derived from a short-for FFQ assess dietary quality in UK adult population surveys? *Public Health Nutrition*, 19 (16), 2915-2923.
59. Braun, V., Clarke, V., 2014. What can “ thematic analysis ” offer health and wellbeing researchers? *Int J Qual Stud Health Well-Being*, 9 (26152), 1-2.
60. Staniszewska, S., Brett, J., Simera, I., Seers, K., Mockford, C., Goodlad, S., Altman, D., Moher, D., Barber, R., Denegri, S., Entwistle, A., Littlejohns, P., Morris, C., Suleman, R., Thomas, V., Tysall, C., 2017. GRIPP2 reporting checklists: tools to improve reporting of patient and public involvement in research, *BMJ*, 2017, 358, 1-7.

For peer review only - http://bmjopen.bmj.com/site/about/guidelines.xhtml

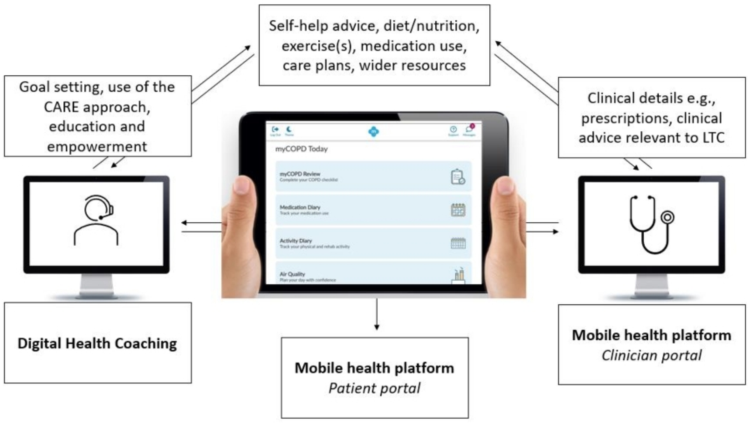

297x210mm (300 x 300 DPI)

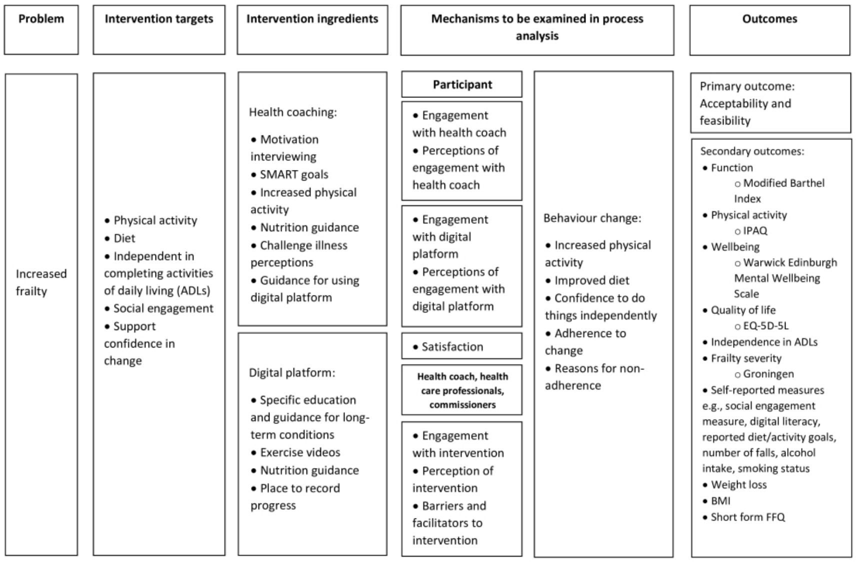

Figure 2: Proposed logic model

297x210mm (300 x 300 DPI)

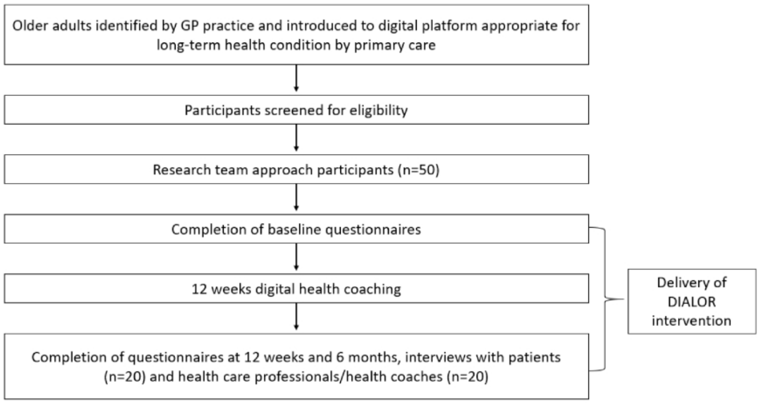

297x210mm (300 x 300 DPI)

SPIRIT

STANDARD PROTOCOL ITEMS: RECOMMENDATIONS FOR INTERVENTIONAL TRIALS

SPIRIT 2013 Checklist: Recommended items to address in a clinical trial protocol and related documents*

Section/item	Item No	Description	Addressed on page number
**Administrative information**			
Title	1	Descriptive title identifying the study design, population, interventions, and, if applicable, trial acronym	1____________
Trial registration	2a	Trial identifier and registry name. If not yet registered, name of intended registry	1____________
	2b	All items from the World Health Organization Trial Registration Data Set	nil___________
Protocol version	3	Date and version identifier	1____________
Funding	4	Sources and types of financial, material, and other support	11___________
Roles and responsibilities	5a	Names, affiliations, and roles of protocol contributors	11___________
	5b	Name and contact information for the trial sponsor	11___________
	5c	Role of study sponsor and funders, if any, in study design; collection, management, analysis, and interpretation of data; writing of the report; and the decision to submit the report for publication, including whether they will have ultimate authority over any of these activities	11___________
	5d	Composition, roles, and responsibilities of the coordinating centre, steering committee, endpoint adjudication committee, data management team, and other individuals or groups overseeing the trial, if applicable (see Item 21a for data monitoring committee)	5,6,9,10______

For peer review only

**Introduction**			
Background and rationale	6a	Description of research question and justification for undertaking the trial, including summary of relevant studies (published and unpublished) examining benefits and harms for each intervention	3_____________
	6b	Explanation for choice of comparators	______________
Objectives	7	Specific objectives or hypotheses	4,5___________
Trial design	8	Description of trial design including type of trial (eg, parallel group, crossover, factorial, single group), allocation ratio, and framework (eg, superiority, equivalence, noninferiority, exploratory)	6_____________
**Methods: Participants, interventions, and outcomes**			
Study setting	9	Description of study settings (eg, community clinic, academic hospital) and list of countries where data will be collected. Reference to where list of study sites can be obtained	6_____________
Eligibility criteria	10	Inclusion and exclusion criteria for participants. If applicable, eligibility criteria for study centres and individuals who will perform the interventions (eg, surgeons, psychotherapists)	7_____________
Interventions	11a	Interventions for each group with sufficient detail to allow replication, including how and when they will be administered	5,6___________
	11b	Criteria for discontinuing or modifying allocated interventions for a given trial participant (eg, drug dose change in response to harms, participant request, or improving/worsening disease)	n/a____________
	11c	Strategies to improve adherence to intervention protocols, and any procedures for monitoring adherence (eg, drug tablet return, laboratory tests)	n/a____________
	11d	Relevant concomitant care and interventions that are permitted or prohibited during the trial	n/a____________
Outcomes	12	Primary, secondary, and other outcomes, including the specific measurement variable (eg, systolic blood pressure), analysis metric (eg, change from baseline, final value, time to event), method of aggregation (eg, median, proportion), and time point for each outcome. Explanation of the clinical relevance of chosen efficacy and harm outcomes is strongly recommended	8,9____________
Participant timeline	13	Time schedule of enrolment, interventions (including any run-ins and washouts), assessments, and visits for participants. A schematic diagram is highly recommended (see Figure)	8_____________

For peer review only - http://bmjopen.bmj.com/site/about/guidelines.xhtml

Sample size	14	Estimated number of participants needed to achieve study objectives and how it was determined, including clinical and statistical assumptions supporting any sample size calculations	6____________
Recruitment	15	Strategies for achieving adequate participant enrolment to reach target sample size	6____________

Methods: Assignment of interventions (for controlled trials)

Allocation:

Sequence generation	16a	Method of generating the allocation sequence (eg, computer-generated random numbers), and list of any factors for stratification. To reduce predictability of a random sequence, details of any planned restriction (eg, blocking) should be provided in a separate document that is unavailable to those who enrol participants or assign interventions	n/a___________
Allocation concealment mechanism	16b	Mechanism of implementing the allocation sequence (eg, central telephone; sequentially numbered, opaque, sealed envelopes), describing any steps to conceal the sequence until interventions are assigned	n/a___________
Implementation	16c	Who will generate the allocation sequence, who will enrol participants, and who will assign participants to interventions	n/a___________
Blinding (masking)	17a	Who will be blinded after assignment to interventions (eg, trial participants, care providers, outcome assessors, data analysts), and how	n/a___________
	17b	If blinded, circumstances under which unblinding is permissible, and procedure for revealing a participant's allocated intervention during the trial	n/a___________

Methods: Data collection, management, and analysis

Data collection methods	18a	Plans for assessment and collection of outcome, baseline, and other trial data, including any related processes to promote data quality (eg, duplicate measurements, training of assessors) and a description of study instruments (eg, questionnaires, laboratory tests) along with their reliability and validity, if known. Reference to where data collection forms can be found, if not in the protocol	9,10__________
	18b	Plans to promote participant retention and complete follow-up, including list of any outcome data to be collected for participants who discontinue or deviate from intervention protocols	9,10__________

For peer review only - http://bmjopen.bmj.com/site/about/guidelines.xhtml

Data management	19	Plans for data entry, coding, security, and storage, including any related processes to promote data quality (eg, double data entry; range checks for data values). Reference to where details of data management procedures can be found, if not in the protocol	9,10__________
Statistical methods	20a	Statistical methods for analysing primary and secondary outcomes. Reference to where other details of the statistical analysis plan can be found, if not in the protocol	9,10__________
	20b	Methods for any additional analyses (eg, subgroup and adjusted analyses)	9,10__________
	20c	Definition of analysis population relating to protocol non-adherence (eg, as randomised analysis), and any statistical methods to handle missing data (eg, multiple imputation)	______________
**Methods: Monitoring**			
Data monitoring	21a	Composition of data monitoring committee (DMC); summary of its role and reporting structure; statement of whether it is independent from the sponsor and competing interests; and reference to where further details about its charter can be found, if not in the protocol. Alternatively, an explanation of why a DMC is not needed	nil___________
	21b	Description of any interim analyses and stopping guidelines, including who will have access to these interim results and make the final decision to terminate the trial	nil__________
Harms	22	Plans for collecting, assessing, reporting, and managing solicited and spontaneously reported adverse events and other unintended effects of trial interventions or trial conduct	6_____________
Auditing	23	Frequency and procedures for auditing trial conduct, if any, and whether the process will be independent from investigators and the sponsor	nil___________
**Ethics and dissemination**			
Research ethics approval	24	Plans for seeking research ethics committee/institutional review board (REC/IRB) approval	n/a__________
Protocol amendments	25	Plans for communicating important protocol modifications (eg, changes to eligibility criteria, outcomes, analyses) to relevant parties (eg, investigators, REC/IRBs, trial participants, trial registries, journals, regulators)	n/a__________

For peer review only - http://bmjopen.bmj.com/site/about/guidelines.xhtml

Consent or assent	26a	Who will obtain informed consent or assent from potential trial participants or authorised surrogates, and how (see Item 32)	6_____________
	26b	Additional consent provisions for collection and use of participant data and biological specimens in ancillary studies, if applicable	6_____________
Confidentiality	27	How personal information about potential and enrolled participants will be collected, shared, and maintained in order to protect confidentiality before, during, and after the trial	9,10__________
Declaration of interests	28	Financial and other competing interests for principal investigators for the overall trial and each study site	11____________
Access to data	29	Statement of who will have access to the final trial dataset, and disclosure of contractual agreements that limit such access for investigators	9,10__________
Ancillary and post-trial care	30	Provisions, if any, for ancillary and post-trial care, and for compensation to those who suffer harm from trial participation	n/a___________
Dissemination policy	31a	Plans for investigators and sponsor to communicate trial results to participants, healthcare professionals, the public, and other relevant groups (eg, via publication, reporting in results databases, or other data sharing arrangements), including any publication restrictions	2___________
	31b	Authorship eligibility guidelines and any intended use of professional writers	nil____________
	31c	Plans, if any, for granting public access to the full protocol, participant-level dataset, and statistical code	nil____________
**Appendices**			
Informed consent materials	32	Model consent form and other related documentation given to participants and authorised surrogates	supplementary_
Biological specimens	33	Plans for collection, laboratory evaluation, and storage of biological specimens for genetic or molecular analysis in the current trial and for future use in ancillary studies, if applicable	Supplementary_

*It is strongly recommended that this checklist be read in conjunction with the SPIRIT 2013 Explanation & Elaboration for important clarification on the items. Amendments to the protocol should be tracked and dated. The SPIRIT checklist is copyrighted by the SPIRIT Group under the Creative Commons "Attribution-NonCommercial-NoDerivs 3.0 Unported" license.

For peer review only - http://bmjopen.bmj.com/site/about/guidelines.xhtml
